# Sham Acupressure Controls Used in Randomized Controlled Trials: A Systematic Review and Critique

**DOI:** 10.1371/journal.pone.0132989

**Published:** 2015-07-15

**Authors:** Jing-Yu Tan, Lorna K. P. Suen, Tao Wang, Alexander Molassiotis

**Affiliations:** 1 School of Nursing, The Hong Kong Polytechnic University, Hung Hom, Kowloon, Hong Kong SAR, China; 2 School of Nursing, Fujian University of Traditional Chinese Medicine, Fuzhou, Fujian, China; 3 The Second Affiliated People’s Hospital, Fujian University of Traditional Chinese Medicine, Fuzhou, Fujian, China; Institute of Tropical Medicine (NEKKEN), Nagasaki University, JAPAN

## Abstract

**Objectives:**

To explore the commonly utilized sham acupressure procedures in existing acupressure trials, and to assess whether different types of sham interventions yield different therapeutic outcomes, and, as far as possible, to identify directions for the future development of an adequate sham acupressure method.

**Methods:**

Randomized controlled trials comparing true acupressure with sham interventions were included. Thirteen electronic databases were adopted to locate relevant studies from inception to July 3, 2014. Meanwhile, eight Chinese journals on complementary and alternative medicine were manually searched to locate eligible articles. In addition, eligible studies listed in the reference lists of the included papers and other related systematic reviews on acupressure were also screened to further search any potentially eligible trials. Methodological quality of the included studies was evaluated using the risk of bias assessment tool developed by the Cochrane Back Review Group. Descriptive analysis was adopted to summarize the therapeutic outcomes.

**Results:**

Sixty-six studies with 7265 participants were included. Methodological quality of the included trials was generally satisfactory. Six types of sham acupressure approaches were identified and “non-acupoint” stimulation was the most frequently utilized sham point while an acupressure device was the most commonly used approach for administering sham treatments. Acupressure therapy was a beneficial approach in managing a variety of health problems and the therapeutic effect was found to be more effective in the true acupressure groups than that in the sham comparative groups. No clear association could be identified between different sham acupressure modalities and the reported treatment outcomes.

**Conclusions:**

A great diversity of sham acupressure controls have been used in clinical practice and research. A solid conclusion whether different sham alternatives are related to different treatment outcomes cannot be derived because of significant clinical heterogeneity among the analyzed trials. Non-acupoints are generally recommended but the definite locations should be identified with caution. For studies using single sham acupoints on hands or legs, it is suggested to apply identical acupressure devices on the same acupoint as in the active intervention without any stimulation. While for studies on pain, stimulation of sham acupoints should be avoided.

## Introduction

Randomized controlled trial (RCT) is one of the commonly used experimental methods for testing the effectiveness of an intervention [1]. To distinguish the specific effect of a therapeutic approach from the non-specific effect, a placebo control is usually employed [2]. Placebo is defined as “any therapy or component of therapy used for its nonspecific, psychological, or psychophysiological effect, or that is used for its presumed specific effect, but is without specific activity for the condition being treated” [3] (p. 371). A placebo intervention is commonly used in experimental drug studies where the “placebo drug” is identical to the active agent without any specific pharmacological activity against the disease. Theoretically, placebo comparisons should be indistinguishable from the true intervention, and most importantly, should be inert, which means only create non-specific physiological and psychological changes [4]. However, an adequate placebo design becomes difficult to implement when a study is adopting complex non-pharmacological interventions such as physiotherapy, acupuncture or acupressure, etc. Multiple mechanisms involved in these types of treatments make it complicated to develop an appropriate placebo control group.

Acupressure has been widely applied in dealing with a variety of health issues globally. In addition to the specific therapeutic effects, stimulation at the acupoints is also believed to generate some non-specific effects, in both physiological and psychological aspects [5]. Placebo controls adopted in acupressure trials are usually referred to as “sham interventions”, which indicate faked acupressure approaches. Various types of sham acupressure have been reported in the literature which mainly differ in three aspects, the selected acupoints, the acupressure approach, and the acupressure intensity. Acupoints adopted in sham procedures commonly include non-acupoints, true acupoints as the active acupressure group, and non-therapeutic acupoints. Non-acupoints generally refer to ineffective body points which cannot be found on established acupuncture-point charts, while non-therapeutic acupoints means irrelevant true acupoints considered to be ineffective for the targeted health problem [2,6].

Sham procedures used in acupuncture have received considerable attention in research. A number of RCTs and systematic reviews have been conducted to investigate the specific treatment effect of acupuncture in a wide range of disorders, especially in pain management, and have yielded contradictory results. Some of them supported the specific benefit of acupuncture [5,7–9], while others argued that the effects of true acupuncture were similar to that in the sham intervention [10–12], and consequently reached the conclusion that the so-called treatment effect of acupuncture may be only a non-specific physiological effect and/or a placebo effect. However, before developing such conclusions, those studies must have a precondition that the employed sham procedure is inert. Similar situations were also detected in studies of acupressure. Despite the significant amounts of research on acupressure during the past decades, it is still uncertain which type of sham acupressure is the most adequate design, and to our knowledge, no study has been conducted so far to summarize the frequently used sham acupressure modalities and to explore their strengths and drawbacks. Hence, the aims of this study are to identify the commonly utilized sham procedures in existing acupressure trials, to assess whether different types of sham intervention yield different therapeutic outcomes, and, if possible, to identify directions for the future development of an adequate sham acupressure mode.

## Methods

This study is a systematic review of the literature **(S1 File)**. A review protocol was created by the first author and it was approved by two experts with experience in acupressure before the commencement of the study **(S2 File)**.

### Data Sources and Search Strategies

Both electronic and manual searches were used to locate relevant studies. Thirteen electronic databases were accessed including PubMed, EMBase, Cochrane Central Register of Controlled Trials (CENTRAL), CINAHL, Allied and Complementary Medicine (AMED), PsycINFO, Thomson Reuters Web of Science, Science Direct, Foreign Medical Journal Service (FMJS), China National Knowledge Infrastructure (CNKI), WanFang Data, Chinese Scientific Journal Database (CQVIP) and Chinese Biomedical Literature Database (CBM), from inception to July 3, 2014. There was no language restriction set for electronic searches. Meanwhile, eight Chinese journals on complementary and alternative medicine (issues published within the latest three years) were manually searched to possibly identify some latest publications which were still not included in the online databases. In addition, eligible studies listed in the reference lists of the included papers and other related systematic reviews on acupressure were also screened to further search any potentially eligible trials. Literature searches were performed independently by two reviewers with Chinese medicine background (JYT and TW). Mesh terms, entry terms, key words and free words such as “acupressure”, “acupress*”, “wristband*” “shiatsu” and “chih ya” were used in the searching strategies. Four selected English and Chinese search strategies are listed in **S1 Table**.

### Inclusion and Exclusion Criteria

RCTs comparing true acupressure with a sham intervention were included. Both true and sham acupressure can be manipulated through either manual intervention or acupressure devices (e.g., wristbands or attached acupressure beads, etc.). To eliminate the effect of electrical stimulation on the therapeutic outcomes of acupressure, studies on electronic acupressure devices were excluded. At the same time, studies on other modalities of acupuncture-point stimulation such as manual/electronic/laser acupuncture and moxibustion, as well as all kinds of auricular therapy were excluded. Studies published in languages other than English and Chinese were excluded.

### Methodological Quality Evaluation

Methodological quality of the included studies was evaluated using the risk of bias assessment tool developed by the Cochrane Back Review Group [13]. The tool can be viewed as an extension of the original criteria recommended by the Cochrane Handbook. It not only includes all the necessary items described in the latest version of the Cochrane risk of bias tool, but also provides more detailed criteria for assessing “incomplete outcome data” and “other sources of potential bias”. The tool consists of 12 items including: (1) “Was the method of randomization adequate?”; (2) “Was the treatment allocation concealed?”; (3) “Was the patient blinded to the intervention?”; (4) “Was the care provider blinded to the intervention?”; (5) “Was the outcome assessor blinded to the intervention?”; (6) “Was the drop-out rate described and acceptable?”; (7) “Were all randomized participants analyzed in the group to which they were allocated?”; (8) “Are reports of the study free of suggestion of selective outcome reporting?”; (9) “Were the groups similar at baseline regarding the most important prognostic indicators?”; (10) “Were co-interventions avoided or similar?”; (11) “Was the compliance acceptable in all groups?” and (12) “Was the timing of the outcome assessment similar in all groups?” [13]. Each item can be rated as of “yes”, “no” or “unclear” where “yes” indicates a low risk of bias [13]. Importantly, different from the original Cochrane risk of bias criteria, the tool used in this study can provide an overall methodological quality assessment for a single trial. The overall quality can be assessed as “low risk of bias” when at least six items are scored as “yes” and no serious methodological flaws are identified (e.g. 80% dropout rate found in one study arm) [13]. In this review, only RCTs with low risk of bias were eligible for final analysis.

### Data Extraction

For each of the included studies, the following data was extracted independently by the same two reviewers: (1) study general information (first author, year of publication, full name of journal, country of origin, type of study design, and study setting); (2) participant characteristics (age, gender, sample size, dropout rate, diagnostic criteria, inclusion and exclusion criteria, and reason for acupressure); (3) true/sham acupressure protocols (practitioner, acupressure equipment, selected acupoints, treatment duration and number of sessions, and acupressure intensity and frequency, etc.); (4) main outcome(s) and results of the therapeutic effects; (5) adverse events associated with acupressure; and (6) assessment of methodological quality (12 items of the risk of bias tool plus examination of the credibility of blinding). For studies comprising more than two active treatment arms, only data from true and sham acupressure arms were extracted for analysis. Meanwhile, for studies including another control group with standard methods of care, data from the standard care arm were also extracted accordingly. Disagreement between the two reviewers was resolved by discussion or by consulting a third reviewer (AM).

### Classification of Sham Acupressure Methods

After going through all of the sham acupressure protocols of the included studies as well as reviewing the sham control classifications for body and auricular acupuncture in previous studies [2,14,15], sham methods in this study were classified into six types which are described in **Table 1**.

**Table 1:** Classification of Sham Acupressure Methods.

Types of Sham Methods	Description
**Type 1**	Sham Acupressure at Non-acupoints by Manual Pressure
**Type 2**	Sham Acupressure at Non-acupoints by Employing Acupressure Devices
**Type 3**	Pseudo-intervention at the Same Acupoints as True Treatment Arm by Manual Light-Touch
**Type 4**	Pseudo-intervention at the Same Acupoints as True Treatment Arm by using Placebo Devices
**Type 5**	Manual Acupressure at Non-Therapeutic (Irrelevant) Acupoints
**Type 6**	Sham Acupressure at Non-Therapeutic (Irrelevant) Acupoints by Adopting Acupressure Devices

### Data Analysis

A quantitative synthesis of the main outcomes was originally proposed in the study protocol. However, a precise meta-analysis was deemed impossible due to the significant clinical heterogeneity identified in the health conditions, patient characteristics, intervention protocols and outcome measures among studies. A subgroup meta-analysis for the same health conditions within each sham acupressure type was also considered but finally abandoned for the reasons that clinical heterogeneity was still considerable within each sham modality, and the number of trials in some sham types was insufficient for data synthesis. To provide an exploratory analysis of the study outcomes, responder rate and responder rate ratio (responder rate in true acupressure group/responder rate in sham group) for each study presenting dichotomous data were calculated, and a risk ratio (RR) with 95% confidence interval (CI) was used to present the responder rate ratio. Descriptive analysis was also used to summarize the therapeutic outcomes of acupressure, for both the overall effect and the subgroup effect by each sham alternative. The overall assessment was to investigate whether true acupressure is superior to sham comparisons while the subgroup analysis was used to assess whether different types of sham methods could induce different therapeutic effects.

Results of the treatment effect for each main outcome in each individual study were extracted by two reviewers according to the following vote counts [2]: “++”: true acupressure is significantly better than sham control for the main outcome; “+”: trend of the main outcome in favor of true acupressure but without statistical significance; “**0”**: no difference between true and sham acupressure; “−”:trend of the main outcome in favor of sham acupressure but without statistical significance; and “− −”: sham intervention is significantly better than true acupressure for the main outcome. For studies reporting multiple main outcomes, all such outcomes were included for analysis. For studies not indicating the main outcomes, all variables with between-group comparison were extracted accordingly.

In addition, subgroup descriptive analyses for different types of health problems, intervention duration and frequency as well as acupressure intensity were also considered when data were available. Based on the analyzed trials, length of treatment was categorized into “extremely short-term” (less than one hour), “short-term” (more than one hour but less than one day), “mid-term” (more than one day but less than one week), and “long-term” (more than one week). For studies on postoperative issue (e.g. nausea, vomiting and pain, etc.) which employed multiple assessment time points, postoperative data within the first 24 hours were abstracted for analysis. The chi-square test was used to assess the difference in dropout rates between the true and sham acupressure arms.

## Results

### Characteristics of the Included Studies

**Fig 1:**
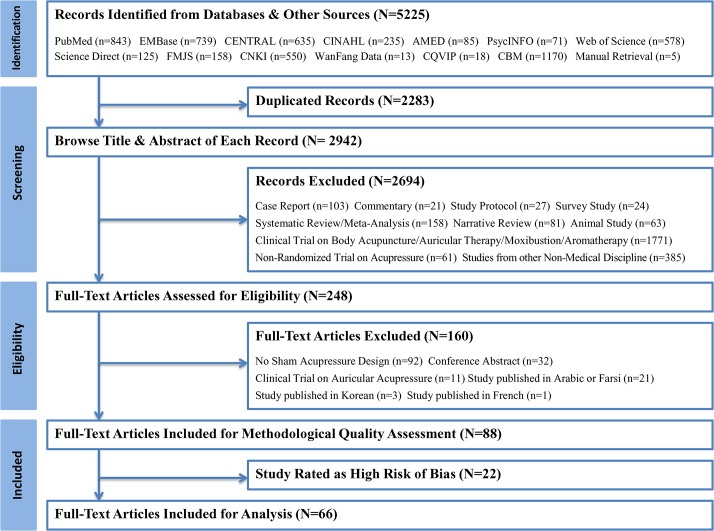
Flow Chart of Study Selection. CENTRAL: Cochrane Central Register of Controlled Trials, CINAHL: Cumulative Index to Nursing and Allied Health Literature, AMED: Allied and Complementary Medicine, FMJS: Foreign Medical Journal Service, CNKI: China National Knowledge Infrastructure, CQVIP: Chinese Scientific Journal Database, CBM: Chinese Biomedical Literature Database

A total of 5225 items were located. Among those 2283 duplicated articles were excluded after screening by the reference management software NoteExpress. A total of 2694 records were subsequently removed by checking titles and abstracts. For the remaining 248 items, full-texts were accessed for evaluating eligibility, among which, 160 were further removed because they failed to include a sham control group (n = 92), were conference proceedings (n = 32), were auricular acupressure studies (n = 11), and were articles published in Arabic/Farsi (n = 21), Korean (n = 3) and French (n = 1). The remaining 88 articles were further adopted for methodological quality evaluation, of which, 22 with high risk of bias were removed (**S2 Table**) and 66 studies [16–81] were finally included for analysis. **Fig 1** shows the flow chart of the study selection.

The included studies comprised 7265 subjects, with an average sample size of 110 patients per study. These studies were published between 1991 and 2015 and were conducted in 18 different countries or regions. Of which, 13 studies originated from Iran, 12 from the United States, ten from Taiwan, five from Sweden, four from the United Kingdom, three from Austria, three from South Korea, three from Canada, two from Italy, two from Ireland, two from India, and one each from Hong Kong, Denmark, Australia, Turkey, Pakistan, Poland and Norway. More than half were carried out in hospitals or medical centers. Acupressure was used to deal with a variety of health problems including nausea and vomiting, labor pain, primary dysmenorrhea, sleep disturbance, postoperative gastrointestinal dysfunction, and anxiety, etc. Characteristics of the included studies can be seen in **Table 2**.

**Table 2:** Characteristics of the Included Studies.

Study & Setting(s)	Condition(s)	Sample Size & Dropout (N/n)	Intervention & Control Groups	Main Outcome(s)	Therapeutic Effects of Acupressure*	Acupressure-related AEs
**Type 1—Sham Acupressure at Non-Acupoints: Manual Acupressure**						
**Tang et al. 2014** [16], Pulmonary Wards of A Medical Center, Taiwan	Cancer-related Fatigue	**Total**: 57/12, **TAG 1**: 24/8, **TAG 2**: 17/2, **SAG**: 16/2	**TAG 1**: Manual Acupressure at True Acupoints, **TAG 2:** Manual Acupressure at True Acupoints + Essential Oils, **SAG:** Manual Acupressure at Non-Acupoints	Fatigue (Tang Fatigue Rating Scale)	**+** ^**1**^	Not Reported^**2**^
**Atrian et al. 2013** [17], Dormitories of Kashan University of Medical Sciences, Iran	Primary Dysmenorrhea	**Total**: 67/8, **TAG**: 33/6, **SAG**: 34/2	**TAG**: Manual Acupressure at True Acupoints, **SAG**: Manual Acupressure at Non-Acupoints	Pain Intensity (VAS)	**0**	No AEs Found in the Study
**Sehhatie-Shafaie et al. 2013** [18], The Public Hospitals of Ardebil, Iran	Labor Pain in Nulliparous Women	**Total**: 84/0, **TAG**: 42/0, **SAG**: 42/0	**TAG**: Manual Acupressure at True Acupoints, **SAG**: Manual Acupressure at Non-Acupoints	Pain Intensity (Visual Pain Scale)	**++**	Not Reported^**2**^
**Chao et al. 2013** [19], An Urban Medical Center in Taipei, Taiwan	Postoperative Gastrointestinal Function	**Total**: 66/6, **TAG**: 30, **SAG**: 30	**TAG**: Manual Acupressure at True Acupoints + Routine Care, **SAG**: Manual Acupressure at Non-Acupoints + Routine Care	Frequency of Bowel Sounds, Time to the First Flatus Passage and Defecation, and Time to Oral Liquid and Solid Intake	**++**(For Bowel Sounds, Flatus Passage, and Liquid Intake), **+** (For Solid Intake and Defecation)	Not Reported^**2**^
**McFadden et al. 2012** [20], University of Colorado at Boulder, United States	Stress Reduction	**Total**: 109/NR, **TAG**: 39/NR, **SAG**: 40/NR, **CG**:30/NR	**TAG**: Manual Acupressure at True Acupoints, **SAG**: Manual Acupressure at Non-Acupoints, **CG**: An Audio Relaxation CD	Stress Responses (Heart Rate, Heart Rate Variability, Skin Conductance Response, State Anxiety Inventory, and Psychological Stress Measure)	**0**	Not Reported^**2**^
**Valiee et al. 2012** [21], Surgery Wards, Hospital of Tehran University of Medical Science, Iran	Preoperative Anxiety	**Total**: 70/0, **TAG**: 35/0, **SAG**: 35/0	**TAG**: Manual Acupressure at True Acupoints, **SAG**: Manual Acupressure at Non-Acupoints	Preoperative Anxiety (VAS), and Vital Signs (Blood Pressure, Heart Rate, and Respiratory Rate)	**++** (For Anxiety, Respiratory Rate, and Systolic Blood Pressure), **+** (For Heart Rate and Diastolic Blood Pressure)	Not Reported^**2**^
**Rad et al. 2012** [22], Rouhani Hospital of Babol University of Medical Science, Iran	Nausea and Vomiting in Pregnancy	**Total**: 85/5, **TAG**: 43/3, **SAG**: 42/2	**TAG**: Manual Acupressure at True Acupoints, **SAG**: Manual Acupressure at Non-Acupoints	Intensity of Nausea (VAS) and Vomiting (Frequency of Vomiting)	**++**	No AEs Found in the Study
**Chang et al. 2011** [23], A Urogynecology Clinic of Queen Elizabeth Hospital, Hong Kong	Urodynamic Stress Incontinence	**Total**: 81/4, **TAG**: 27/1, **SAG**: 27/1, **CG**: 27/2	**TAG**: Manual Acupressure at True Acupoints + Pelvic Floor Muscle Training, **SAG**: Manual Acupressure at Non-Acupoints + Pelvic Floor Muscle Training, **CG**: Pelvic Floor Muscle Training	Pelvic Muscle Strength (Perineometry through Measuring Vaginal Squeeze Pressure)	**++**, TAG was Significantly Better than SAG and CG; No Difference was Found between SAG and CG	Not Reported^**2**^
**McFadden et al. 2011** [24], A Community in Colorado, United States	Traumatic Brain Injury	**Total**: 42/0, **TAG**: 21/0, **SAG**: 21/0	**TAG**: Manual Acupressure at True Acupoints, **SAG**: Manual Acupressure at Non-Acupoints	Cognitive Impairment and State of Being following Traumatic Brain Injury	**++**	Not Reported^**2**^
**Kashefi et al. 2011** [25], Bojnoord University of Medical Science, Iran	Women’s General Health	**Total**: 86/10, **TAG**: 43/6, **SAG**: 43/4	**TAG**: Manual Acupressure at True Acupoints, **SAG**: Manual Acupressure at Non-Acupoints	Women’s General Health (General Health Questionnaire)	**++**	No AEs Found in the Study
**Reza et al. 2010** [26], Kahrizak Charity Nursing Home, Iran	Sleep Disturbances	**Total**: 90/13, **TAG**: 30/5, **SAG**: 30/4, **CG**: 30/4	**TAG**: Manual Acupressure at True Acupoints + Usual Care, **SAG**: Manual Acupressure at Non-Acupoints + Usual Care, **CG**: Usual Care	Self-Reported Sleep Habits (Pittsburgh Sleep Quality Index)	**++**, TAG was Significantly Better than SAG and CG; The Sleep Quality in SAG was Better than That in CG but There was no Statistical Significance	Not Reported^**2**^
**McFadden & Hernández 2010** [27], Denver/Boulder Community in Colorado, United States	Cardiovascular Function in Stroke Survivors	**Total**: 16/3, **TAG**: 7/1, **SAG**: 9/2	**TAG**: Manual Acupressure at True Acupoints, **SAG**: Manual Acupressure at Non-Acupoints	Heart Rate and Blood Pressure	**++** (For Heart Rate), **0** (For Blood Pressure)	Not Reported^**2**^
**Maa et al. 2007** [28], Department of Thoracic Medicine, Chang Gung Memorial Hospital, Taiwan	Symptoms and Health-Related Quality of Life in Bronchiectasis Patients	**Total**: 49/14, **TAG**: 16/5, **SAG**: 17/6, **CG**: 16/3	**TAG**: Manual Acupressure at True Acupoints + Standard Care, **SAG**: Manual Acupressure at Non-Acupoints + Standard Care, **CG**: Standard Care	Daily Sputum Amounts, Efforts to clean Secretions (Sputum Self-Assessment), Six-Minute Walking Distance, Breathing Difficulty, and Quality of Life (SGRQ)	**0**, TAG was Better than CG Regarding to the SGRQ Activity Domain, and SAG was better than CG in the Improvement of Sputum Self-Assessment	Not Reported^**2**^
**Shin et al. 2007** [29], Two General Hospitals, South Korea	Nausea, Vomiting, and Ketonuria Levels in Women with Hyperemesis Gravidarum	**Total**: 66/NR, **TAG**: 23/NR, **SAG**: 21/NR, **CG**: 22/NR	**TAG**: Manual Acupressure at True Acupoints + Routine Intravenous Therapy, **SAG**: Manual Acupressure at Non-Acupoints + Routine Intravenous Therapy, **CG**: Routine Intravenous Treatment	Degree of Nausea and Vomiting (The Rhodes Index of Nausea, Vomiting, and Retching), and Degree of Ketonuria	**++**, The Degree of Nausea and Vomiting in TAG was Statistically Lower than SAG and CG, and No Difference was Found between SAG and CG	Not Reported^**2**^
**Bertalanffy et al. 2004** [30], Sites of Accident, Austria	Motion Sickness in Patients with Trauma	**Total**: 100/0, **TAG**: 50/0, **SAG**: 50/0	**TAG**: Manual Acupressure at True Acupoints, **SAG**: Manual Acupressure at Non-Acupoints	Nausea Intensity (VAS)	**++**	Not Reported^**2**^
**Chen et al. 2003** [31], A Mid-Taiwan Teaching Hospital, Taiwan	Gastrointestinal Motility after Surgery	**Total**: 41/0, **TAG**: 21/0, **SAG**: 20/0	**TAG**: Manual Acupressure at True Acupoints, **SAG**: Manual Acupressure at Non-Acupoints	Gastrointestinal Motility (Multifunctional Stethoscope)	**++**	Not Reported^**2**^
**Tsay & Chen 2003** [32], Four Dialysis Centers in Major Hospitals in Taipei, Taiwan	Sleep Quality	**Total**: 105/7, **TAG**: NR, **SAG**: NR, **CG**: NR	**TAG**: Manual Acupressure at True Acupoints + Usual Care, **SAG**: Manual Acupressure at Non-Acupoints + Usual Care, **CG**: Usual Care	Sleep Quality (Pittsburgh Sleep Quality Index)	**0**, TAG was Significantly Better than CG; No Difference was Found between SAG and CG	Not Reported^**2**^
**Kober et al. 2002** [33], Sites of Accident, Austria	Pre-Hospital Analgesia	**Total**: 60/0, **TAG**: 19/0, **SAG**: 20/0, **CG**: 21/0	**TAG**: Manual Acupressure at True Acupoints, **SAG**: Manual Acupressure at Non-Acupoints, **CG**: No Acupressure	Pain (VAS), Anxiety (VAS), and Heart Rate	Comparisons between Groups were not Performed (or Not Clearly Reported)	Not Reported^**2**^
**Belluomini et al. 1994** [34], Department of Obstetrics and Gynecology, California Pacific Medical Center, United States	Nausea and Vomiting in Pregnancy	**Total**: 90/30, **TAG**: 46/16, **SAG**: 44/14	**TAG**: Manual Acupressure at True Acupoints, **SAG**: Manual Acupressure at Non-Acupoints	Nausea and Vomiting (The Index of Nausea, Vomiting, and Retching)	**++** (For Nausea), **+** (For Vomiting)	Not Reported^**2**^
**Type 2—Sham Acupressure at Non-Acupoints: Acupressure Bands or Other Devices**						
**Adib-Hajbaghery & Etri, 2013** [35], A General Surgical Ward of A University Hospital, Iran	Postoperative Pain, Nausea, and Vomiting	**Total**: 70/0, **TAG**: 35/0, **SAG**: 35/0	**TAG**: Acupressure at True Acupoints using Acupressure Band, **SAG**: Acupressure at Non-Acupoints using Acupressure Band	Severity of Nausea and Pain (VAS), and Severity of Vomiting (Frequency of Vomiting)	**++** (For Pain), **0** (For Nausea), **NA** (For Vomiting)^**3**^	Not Reported^**2**^
**Alessandrini et al. 2012** [36], Otolaryngology Department, University of Rome “Tor Vergata”, Italy	Acute Vertigo	**Total**: 204/0, **TAG**: 102/0, **SAG**: 102/0	**TAG**: Acupressure at True Acupoints using Acupressure Band, **SAG**: Acupressure at Non-Acupoints using Acupressure Band	Severity of Vertigo and Neurovegetative Symptoms (VAS)	Comparisons between Groups were not Performed (or Not Clearly Reported)	Not Reported^**2**^
**Soltani et al. 2011** [37], Farabi Hospital, Iran	Postoperative Nausea and Vomiting	**Total**: 200/0, **TAG**: 50/0, **SAG 1**: 50/0, **SAG 2**: 50/0, **SAG 3**: 50/0	**TAG**: Using Acupressure Wrist Band at True Acupoints, **SAG 1**: Using Acupressure Wrist Band at Non-Acupoints, **SAG 2:** Using Acupressure Wrist Band at Non-Acupoints + Metoclopramide, **SAG 3:** Using Acupressure Wrist Band at Non-Acupoints + Ondansetron	Postoperative Nausea and Vomiting during the First Two Hours (0–2 Hours) and the Following 22 Hours (2–24 Hours) after Surgery	**++** ^**4**^	Not Reported^**2**^
**Bao et al. 2011** [38], The Sidney Kimmel Comprehensive Cancer Center at Johns Hopkins, United States	Pain in Cancer Patients undergoing Bone Marrow Aspiration and Biopsy (BMAB)	**Total**: 78/1, **TAG**: 38/1, **SAG**: 40/0	**TAG**: Acupressure at True Acupoints using Magnetic Acupressure Suction Cups + Standard Local Analgesics, **SAG**: Acupressure at Non-Acupoints using Magnetic Acupressure Suction Cups+ Standard Local Analgesics	Pain Intensity (VAS)	**0**	Mild and Transient Bruising or A Rash at the Acupressure Site (n = 10)
**Majholm & Møller 2011** [39], Copenhagen University Hospital, Denmark	Postoperative Nausea and Vomiting	**Total**: 134/22, **TAG**: 67/8, **SAG**: 67/14	**TAG**: Acupressure at True Acupoints using Acupressure Band, **SAG:** Acupressure at Non-Acupoints using Acupressure Band	Postoperative Nausea and/or Vomiting	**0**	Redness (n = 40), Swelling (n = 17), Tenderness (n = 16), and Paresthesias (n = 4)
**Sinha et al. 2011** [40], A Single Tertiary Maternity Unit, Australia	Nausea and Vomiting during Labour and Delivery	**Total**: 340/11, **TAG**: 170/6, **SAG**: 170/5	**TAG**: Acupressure at True Acupoints using Acupressure Band, **SAG**: Acupressure at Non-Acupoints using Placebo Acupressure Band	Incidence of Nausea and/or Vomiting	**0**	Discomfort from Band (n = 25)
**Wang et al. 2008** [41], A Hospital (Details not Described), United States	Pre-Procedural Anxiety and Intra-Procedural Propofol Needs in Children Undergoing Anesthesia	**Total**: 52/0, **TAG**: 26/0, **SAG**: 26/0	**TAG**: Acupressure at True Acupoints using Acupressure Beads, **SAG**: Acupressure at Non-Acupoints using Acupressure Beads	Anxiety Level (State Anxiety Inventory for Children)	**++**	Not Reported^**2**^
**Turgut et al. 2007** [42], Department of Anesthesiology, Ankara Oncology Hospital, Turkey	Postoperative Nausea and Vomiting	**Total**: 102/2, **TAG**: 51/1, **SAG**: 51/1	**TAG**: Acupressure at True Acupoints using Acupressure Band, **SAG**: Acupressure at Non-Acupoints using Acupressure Band	Severity of Postoperative Nausea and Vomiting	**++**	Erythema and Swelling of the Treated Hand (n = 1)
**Heazell et al. 2006** [43], A Single Secondary Care Center, United Kingdom	Nausea and Vomiting in Early Pregnancy	**Total**: 80/0, **TAG**: 40/0, **SAG**: 40/0	**TAG**: Acupressure at True Acupoints using Acupressure Band, **SAG**: Acupressure at Non-Acupoints using Acupressure Band	No. of Days of Hospital Stay, and No. of Patients Who Required ≧ 4 Days in the Hospital	**0** (For No. of Days of Hospital Stay), **++** (For No. of Patients Who Required ≧ 4 Days in the Hospital)	No Discomforts Found in the Study
**Wang et al. 2005** [44], A Hospital (Details not Described), United States	Preoperative Parental Anxiety	**Total**: 61/NR, **TAG**: 28/NR, **SAG**: 33/NR	**TAG**: Acupressure at True Acupoints using Acupressure Beads, **SAG**: Acupressure at Non-Acupoints using Acupressure Beads	Parental Anxiety (State Anxiety Inventory)	**++**	Not Reported^**2**^
**Alkaissi et al. 2005** [45], Department of Otolaryngology, University Hospital of Linköping, Sweden	Tolerance to Nauseogenic Motion Stimulation	**Total**: 60/0, **TAG**: 20/0, **SAG**: 20/0, **CG**: 20/0	**TAG**: Acupressure at True Acupoints using Acupressure Band, **SAG**: Acupressure at Non-Acupoints using Acupressure Band, **CG**: No Acupressure	Time to the Onset of Moderate Nausea	**+**, Mean Time to Moderate Nausea was Statistically Longer in TAG than That in CG, SCG was also Longer than CG but There was no Statistical Significance	Feeling Uncomfortable and Tight (n = 2), and Swelling of the Hand (n = 1)
**Samad et al. 2003** [46], Aga Khan University Hospital, Pakistan	Postoperative Nausea and Vomiting	**Total**: 50/0, **TAG**: 25/0, **SAG**: 25/0	**TAG**: Acupressure at True Acupoints using Wrist-Band with Plastic Bead, **SAG**: Acupressure at Non-Acupoints using Wrist-Band with Plastic Bead	Incidence of Nausea and Vomiting	**0**	Not Reported^**2**^
**Alkaissi et al. 2002** [47], University Hospital of Linköping, Sweden	Postoperative Nausea and Vomiting	**Total**: 410/30, **TAG**: 135/NR, **SAG**: 139/NR, **CG**: 136/NR	**TAG**: Acupressure at True Acupoints using Acupressure Band + Routine Anesthesia, **SAG**: Acupressure at Non-Acupoints using Acupressure Band + Routine Anesthesia, **CG**: Routine Anesthesia	Complete Response of Postoperative Nausea and Vomiting (No Report of Nausea, Vomiting or Rescue Medication)	**+**,Both TAG and SAG were Better than CG	Discomforts, Red Indentation or Itching (n = 15), Headache and Dizziness (n = 1),Deep Marks, Blistering or Swelling (n = 45)
**Agarwal et al. 2000** [48], Setting not Described, India	Postoperative Nausea and Vomiting	**Total**: 200/0, **TAG**: 100/0, **SAG**: 100/0	**TAG**: Acupressure at True Acupoints using Acupressure Bands, **SAG**: Acupressure at Non-Acupoints using Acupressure Bands	Postoperative Nausea and Vomiting	**+**	No AEs Found in the Study
**Harmon et al. 2000** [49], Rotunda Hospital, Ireland	Nausea and Vomiting during and after Surgery	**Total**: 94/NR, **TAG**: 47/NR, **SAG**: 47/NR	**TAG**: Acupressure at True Acupoints using Acupressure Band, **SAG**: Acupressure at Non-Acupoints using Acupressure Band	Nausea and Vomiting during and after Surgery	**++**	Not Reported^**2**^
**Harmon et al. 1999** [50], Rotunda Hospital, Ireland	Postoperative Nausea and Vomiting	**Total**: 104/0, **TAG**: 52/0, **SAG**: 52/0	**TAG**: Acupressure at True Acupoints using Acupressure Band, **SAG**: Acupressure at Non-Acupoints using Acupressure Band	Postoperative Nausea and Vomiting	**++**	No AEs Found in the Study
**Alkaissi et al. 1999** [51], University Hospital of Linköping, Sweden	Postoperative Nausea and Vomiting	**Total**: 60/10, **TAG**: 20/NR, **SAG**: 20/NR, **CG**: 20/NR	**TAG**: Acupressure at True Acupoints using Acupressure Band + Routine Anesthesia, **SAG**: Acupressure at Non-Acupoints using Acupressure Band + Routine Anesthesia, **CG**: Routine Anesthesia	Complete Response of Postoperative Nausea and Vomiting (No Report of Nausea, Vomiting or Rescue Medication)	**0**, Both TAG and SAG were Better than CG, but There were No Statistical Significances	Not Reported^**2**^
**Fan et al. 1997** [52], Maimonides Medical Center, United States	Postoperative Nausea and Vomiting	**Total**: 200/0, **TAG**: 108/0, **SAG**: 92/0	**TAG**: Acupressure at True Acupoints using Acupressure Band, **SAG**: Acupressure at Non-Acupoints using Acupressure Band	Postoperative Nausea and Vomiting	**++**	No AEs Found in the Study
**O’Brien et al. 1996** [53], Setting Not Described, Canada	Nausea and Vomiting During Pregnancy	**Total**: 161/12, **TAG**: 54/NR, **SAG**: 53/NR, **CG**: 54/NR	**TAG**: Acupressure at True Acupoints using Acupressure Band, **SAG**: Acupressure at Non-Acupoints using Acupressure Band, **CG**: No Acupressure	Nausea and Vomiting (The Rhodes Index of Nausea, Vomiting, and Retching)	**0**, No Difference was Detected Across Groups	Not Reported^**2**^
**Felhendler & Lisander 1996** [54], An Ambulatory Unit in Linköping University Hospital, Sweden	Postoperative Pain	**Total**: 40/NR, **TAG**: 20/NR, **SAG**: 20/NR	**TAG**: Acupressure at True Acupoints using A Dentist’s Tool, **SAG**: Acupressure at Non-Acupoints using A Dentist’s Tool	Postoperative Pain (VAS)	**++**	Not Reported^**2**^
**Bayreuther et al. 1994** [55], Five General Practices in Central Southampton, United Kingdom	Early Morning Sickness	**Total**: 23/8, **TAG**: 11/4, **SAG**: 12/4	**TAG**: Acupressure at True Acupoints using Acupressure Band, **SAG**: Acupressure at Non-Acupoints using Acupressure Band	Nausea (VAS)	**++**	Not Reported^**2**^
**Type 3—Pseudo-Intervention at the Same Acupoints as Intervention Group: Manual Light-Touch without Acupressure**						
**Hamidzadeh et al. 2012** [56], Fatemiyeh Educational and Research Hospital in Shahroud, Iran	Labor Pain	**Total**: 100/0, **TAG**: 50/0, **SAG**: 50/0	**TAG**: Manual Acupressure at True Acupoints, **SAG**: Light Touch on the Same Acupoints	Intensity of Labor Pain (VAS)	**++**	No AEs Found in the Study
**Mirbagher-Ajorpaz et al. 2011** [57], Dormitories of Kashan University of Medical Science, Iran	Primary Dysmenorrhea	**Total**: 30/0, **TAG**: 15/0, **SAG**: 15/0	**TAG**: Manual Acupressure at True Acupoints, **SAG**: Light Touch on the Same Acupoints	Severity of Dysmenorrhea (VAS)	**++**	Not Reported^**2**^
**Sun et al. 2010** [58], Two Long-Term Care Facilities in Northern Taiwan, Taiwan	Insomnia	**Total**: 50/6, **TAG**: 25/2, **SAG**: 25/4	**TAG**: Manual Acupressure at True Acupoints, **SAG**: Light Touch on the Same Acupoints	Insomnia (The Athens Insomnia Scale-Taiwan Form)	**++**	Not Reported^**2**^
**Kashanian & Shahali 2010** [59], Akbarabadi Teaching Hospital in Tehran, Iran	Duration and Pain of the Active Phase of Labor	**Total**: 120/0, **TAG**: 60/0, **SAG**: 60/0	**TAG**: Manual Acupressure at True Acupoints, **SAG**: Light Touch on the Same Acupoints	Severity of Labor Pain (VAS) and Duration of Active Phase	**++**	Not Reported^**2**^
**Hjelmstedt et al. 2010** [60], Sree Avittom Thirunal Hospital in Trivandrum, India	Labor Pain	**Total**: 213/1, **TAG**: 71/0, **SAG**: 71/0, **CG**:71/1	**TAG**: Manual Acupressure at True Acupoints + Usual Care, **SAG**: Light Touch on the Same Acupoints + Usual Care, **CG**: Usual Care	Intensity of Labor Pain (VAS)	**++**, TAG was Significantly Better than CG; SAG was Better than CG but There was no Statistical Significance	Not Reported^**2**^
**Hsu et al. 2006** [61], Two Long-Term Care Facilities, Taiwan	Insomnia	**Total**: 50/0, **TAG**: 25/0, **SAG**: 25/0	**TAG**: Manual Acupressure at True Acupoints, **SAG**: Light Touch on the Same Acupoints	Insomnia (Pittsburgh Sleep Quality Index)	**++**	Not Reported^**2**^
**Lee et al. 2004** [62], Delivery Room in A University Hospital, South Korea	Labor Pain and Length of Delivery Time	**Total**: 89/14, **TAG**: 36, **SAG**: 39	**TAG**: Manual Acupressure at True Acupoints, **SAG**: Light Touch on the Same Acupoints	Severity of Labor Pain (VAS) and Duration of Labor to Delivery	**++**	Not Reported^**2**^
**Type 4—Pseudo-Intervention at the Same Acupoints as Intervention Group: Placebo Acupressure Devices**						
**Nilsson et al. 2015** [63], Department of Neurosurgery of Umeå University Hospital, Sweden	Postoperative Nausea and Vomiting	**Total**: 120/25, **TAG**: 52/9, **SAG**: 68/16	**TAG**: Acupressure at True Acupoints using Acupressure Band, **SAG**: Sham Acupressure at the Same Acupoints using A Placebo Acupressure Band	Postoperative Nausea (NRS)	**0**	Swelling (n = 12), Bruises (n = 2), Paresthesia (n = 1), and Pain (n = 1)
**Molassiotis et al. 2013** [64], Christie NHS Foundation Trust, Clatterbridge Centre for Oncology, Southport General Infirmary, and Several Hospitals, United Kingdom	Chemotherapy-Induced Acute and Delayed Nausea	**Total**: 500/128, **TAG**: 168/36, **SAG**: 166/47, **CG**: 166/45	**TAG**: Acupressure at True Acupoints using Acupressure Band + Standard Antiemetics, **SAG**: Sham Acupressure at the Same Acupoints using A Placebo Acupressure Band+ Standard Antiemetics, **CG**: Standard Antiemetics	Chemotherapy-Induced Nausea (The Nausea Experience Subscale of The Rhodes Index of Nausea, Vomiting, and Retching)	**0**, Nausea Experience was slightly lower in Both TAG and SAG than that in CG, but There was No Statistical Significance	Transient AEs (n = 6): Minor Swelling and Tightness in the Area of Wristbands, and Discomfort when Wearing the Wristbands
**Noroozinia et al. 2013** [65], Imam Khomeini General Hospital of Urmia, Iran	Postoperative Nausea and Vomiting	**Total**: 152/0, **TAG**: 76/0, **SAG**: 76/0	**TAG**: Acupressure at True Acupoints using Acupressure Band, **SAG**: Sham Acupressure at the Same Acupoints using A Placebo Acupressure Band	Postoperative Nausea and Vomiting (VAS)	**++**	Not Reported^**2**^
**Soltanzadeh et al. 2012** [66], Hospital (Details not Described), Iran	Postoperative Nausea and Vomiting	**Total**: 120/0, **TAG**: 40/0, **SAG 1**: 40/0, **SAG 2**: 40/0	**TAG**: Acupressure at True Acupoints using Acupressure Band, **SAG 1**: Sham Acupressure at the Same Acupoints using A Dummy Band, **SAG 2**: Sham Acupressure at the Same Acupoints using A Dummy Band + Metoclopramide	Incidence of Postoperative Nausea and Vomiting	**++** (For Nausea), **+** (For Vomiting)	No AEs Found in the Study
**White et al. 2012** [67], Cedars Sinai Medical Center in Los Angeles, United States	Postoperative Nausea and Vomiting	**Total**: 100/0, **TAG**: 50/0, **SAG**: 50/0	**TAG**: Acupressure at True Acupoints using Acupressure Strip, **SAG**: Sham Acupressure at the Same Acupoints using A Placebo Acupressure Strip	Incidence of Postoperative Nausea and Vomiting	**++**	Constipation (n = 2), Headache (n = 3), Fatigue (n = 6), and Drowsiness (n = 3)^**5**^
**Ho et al. 2006** [68], Taipei Veterans General Hospital, Taiwan	Nausea and Vomiting During Surgery	**Total**: 110/0, **TAG**: 55/0, **SAG**: 55/0	**TAG**: Acupressure at True Acupoints using Acupressure Band, **SAG**: Sham Acupressure at the Same Acupoints using A Placebo Acupressure Band	Incidence of Nausea and Vomiting During Surgery	**+**	No AEs Found in the Study
**Klein et al. 2004** [69], Toronto General Hospital, Canada	Postoperative Nausea and Vomiting	**Total**: 152/0, **TAG**: 75/0, **SAG**: 77/0	**TAG**: Acupressure at True Acupoints using Acupressure Band, **SAG**: Sham Acupressure at the Same Acupoints using A Placebo Acupressure Band	Incidence of Postoperative Nausea and Vomiting	**+**	No AEs Found in the Study
**Schultz et al. 2003** [70], A Tertiary Care Hospital in Northern New England, Portland	Postoperative Nausea and Vomiting	**Total**: 143/40, **TAG 1**:30, **TAG 2**:24, **SAG 1**:24, **SAG 2**:25	**TAG 1**: Acupressure Band at True Acupoints + Droperidol, **TAG 2**: Acupressure Band at True Acupoints + Placebo Drug, **SAG 1**: Placebo Band at the Same Acupoints+ Droperidol, **SAG 2**: Placebo Band at the Same Acupoints+ Placebo Drug	Incidence of Postoperative Nausea and Vomiting	—(For Nausea),—(For Vomiting) ^**6**^	Not Reported^**2**^
**Dent et al. 2003** [71], The Coronary Care Unit of A District Hospital, United Kingdom	Acute Myocardial Infarction-Related Nausea and Vomiting	**Total**: 301/0, **TAG**: 95/0, **SAG**: 98/0, **CG**: 108/0	**TAG**: Acupressure at True Acupoints using Acupressure Band, **SAG**: Sham Acupressure at the Same Acupoints using A Placebo Acupressure Band, **CG**: No Additional Intervention	Incidence of Nausea and Vomiting	**+**,TAG was Better than SAG and CG, No Difference was Found between SAG and CG	Not Reported^**2**^
**Norheim et al. 2001** [72], A University Hospital, Norway	Morning Sickness in Pregnancy	**Total**: 97/13, **TAG**: NR, **SAG**: NR	**TAG**: Acupressure at True Acupoints using Acupressure Band, **SAG**: Sham Acupressure at the Same Acupoints using A Placebo Acupressure Band	Incidence and Duration of Nausea and Vomiting	**+** (For Incidence of Nausea and Vomiting), **++** (For Duration of Nausea and Vomiting)	Pain, Numbness, Soreness, and Swelling (No. not Given); Worse Symptoms (n = 3)
**Steele et al. 2001** [73], Seventeen Medical Clinics or Offices in Southern Michigan, United States	Nausea and Vomiting During Pregnancy	**Total**: 138/28, **TAG**: 85/17, **SAG**: 53/11	**TAG**: Acupressure at True Acupoints using Acupressure Band, **SAG**: Sham Acupressure at the Same Acupoints using A Placebo Acupressure Band	Severity and Frequency of Nausea and Vomiting	**++**	Not Reported^**2**^
**Woods 1999** [74], Malcolm Grow Air Force Medical Center, United States	Postoperative Nausea and Vomiting	**Total**: 10/NR, **TAG**: 5/NR, **SAG**: 5/NR	**TAG**: Acupressure at True Acupoints using Acupressure Band, **SAG**: Sham Acupressure at the Same Acupoints using A Placebo Acupressure Band	Postoperative Nausea and Vomiting (VAS)	-	Not Reported^**2**^
**Duggal et al. 1998** [75], Hospital (Details not Described), Canada	Postoperative Nausea and Vomiting	**Total**: 263/19, **TAG**: 122, **SAG**: 122	**TAG**: Acupressure at True Acupoints using Acupressure Band, **SAG**: Sham Acupressure at the Same Acupoints using A Placebo Acupressure Band	Incidence of Nausea and Vomiting	**+**	Tightness (n = 22), Swollen Hands (n = 43), Infusion Problem (n = 4), and Itching Wrists (n = 9)
**Lewis et al. 1991** [76], Hospital (Details not Described), United States	Postoperative Nausea and Vomiting	**Total**: 66/2, **TAG**: 33/2, **SAG**: 33/0	**TAG**: Acupressure at True Acupoints using Acupressure Band, **SAG**: Sham Acupressure at the Same Acupoints using A Placebo Acupressure Band	Incidence of Postoperative Nausea and Vomiting	**0**	Bands were Well Tolerated
**Type 5—Acupressure at Non-Therapeutic Acupoints (Irrelevant Acupoints): Manual Acupressure**						
**Suh 2012** [77], A University Cancer Center in Seoul, South Korea	Chemotherapy-Induced Nausea and Vomiting	**Total**: 120/15, **TAG 1**:30/5, **TAG 2**:30/1, **SAG**: 30/7, **CG**:30/2	**TAG 1**: Acupressure at Therapeutic Acupoints using Acupressure Band, **TAG 2**: Acupressure at Therapeutic Acupoints using Acupressure Band + Counseling, **SAG**: Manual Acupressure at Irrelevant Acupoints, **CG**: Counseling Only	Chemotherapy-Induced Nausea and Vomiting (The Index of Nausea, Vomiting, and Retching)	**++** (For Acute and Delayed Vomiting) ^**7**^	Not Reported^**2**^
**Lang et al. 2007** [78], A Paramedic-Based Rescue System, Austria	Pre-Hospital Analgesia	**Total**: 32/1, **TAG**: 16/1, **SAG**: 16/0	**TAG**: Manual Acupressure at Therapeutic Acupoints, **SAG**: Manual Acupressure at Irrelevant Acupoints	Intensity of Pain and Anxiety (VAS), and Blood Pressure and Heart Rate	**++** (For Pain, Anxiety and Heart Rate), **0** (Blood Pressure)	Not Reported^**2**^
**Wu et al. 2007** [79], **Wu et al. 2004** [80], A Medical Center and Three Regional Hospitals in Taipei, Taiwan	Chronic Dyspnea in Chronic Obstructive Pulmonary Disease	**Total**: 62/18, **TAG**: 22, **SAG**: 22	**TAG**: Manual Acupressure at Therapeutic Acupoints, **SAG**: Manual Acupressure at Irrelevant Acupoints	Depression (The Geriatric Depression Scale), Anxiety (The State Anxiety Inventory), Chronic Dyspnea (Pulmonary Function Status and Dyspnea Questionnaire Modified and VAS), and Six-Minute Walking Tests	**++**	Not Reported^**2**^
**Type 6—Acupressure at Non-Therapeutic Acupoints (Irrelevant Acupoints): Acupressure Bands or Other Devices**						
**Nordio & Romanelli 2008** [81], Setting not Described, Italy	Insomnia	**Total**: 40/7, **TAG**: 20/2, **SAG**: 20/5	**TAG**: Acupressure at Therapeutic Acupoints using Wrist Acupressure Device, **SAG**: Acupressure at Irrelevant Acupoints using Wrist Acupressure Device	Level of Global Life Quality (General Health Questionnaire), Sleep Quality (Pittsburgh Sleep Quality Index), and Anxiety (State Trait Anxiety Inventory); and Urinary Melatonin Metabolite	**NR** (For Life Quality, Comparison between Groups was not Shown), **++** (For Sleep Quality), **+** (For Anxiety), **++** (For Urinary Melatonin Metabolite)	No AEs Found in the Study

**AE**: Adverse event, **TAG**: True acupressure group, **SAG**: Sham acupressure group, **VAS**: Visual Analogue Scale, **NA**: Not applicable, **CG**: Control group, **SGRQ**: The Saint George Respiratory Questionnaire, **NRS**: Numerical Rating Scale

**1**: For true acupressure group 1 and sham acupressure group

**2**: The study did not assess acupressure-related adverse events

**3**: Statistical analysis was not conducted for vomiting

**4**: For true acupressure group and sham acupressure group 1

**5**: Incidence of postoperative adverse events, not sure whether these events were associated with acupressure

**6**: For true acupressure group 1 and sham acupressure group 1

**7**: For true acupressure group 1 and sham acupressure group.

*Vote counts of results of therapeutic effects: “++”: True acupressure group is significantly better than sham acupressure group for the main outcome; “+”: Trend of the main outcome in favor of true acupressure group but without statistical significance; “**0**”: No difference was found between true and sham acupressure groups; “−”:Trend of the main outcome in favor of sham acupressure group but without statistical significance; “− −”: sham acupressure group is significantly better than true acupressure group for the main outcome.

### Methodological Quality and Risk of Bias of the Included Studies

Results of the risk of bias assessment for all the included trials can be seen in **S3 Table**. Methodological quality of the included trials was generally satisfactory, as already all studies of high risk of bias were excluded. Forty-nine trials adequately described the method of randomization. Twenty-six studies appropriately described the allocation concealment. Blinding of participants was adopted in 45 trials. Outcome assessor was blinded to the intervention in the majority of studies (51 studies), but there were only 17 studies which reported blinding for the care provider. Eighteen studies employed a single-blind design for either participant, or care provider, or outcome assessor. Double-blinding for both participant and outcome assessor was utilized in 26 trials, and blinding for both the care provider and the outcome assessor was used in four studies. There were 11 trials that incorporated a triple-blind design. No trial tested the credibility of blinding during the study period.

Dropout of study subjects was mentioned and acceptable in 89.4% of the included studies. Thirty-two trials reported that all randomized participants were included for analysis. Selective outcome reporting was detected in only one study, and baseline data were similar between groups in most of the studies. Co-intervention (e.g. intra-operative anesthesia or standard care, etc.) was similar or avoidable in more than half of the articles (42 studies). Participants’ compliance to treatment was mentioned and satisfactorily achieved in seven studies. The timing of outcome assessment was found similar among groups in 96.97% of the included trials.

### Description of Acupressure Protocols

True acupressure protocols used in the included trials are presented in **Table 3**. Among the analyzed trials, 30 adopted manual acupressure while another 36 chose acupressure equipment to provide constant pressure on the targeted acupoints. The majority (52 studies) described the person conducting acupressure, being an acupuncturist, acupressure practitioner, anesthetist, nurse, paramedic and study investigator, etc. For studies where acupressure was administered by non-acupuncture/acupressure professionals, 60% (27/45) specified that the intervention operator had received training in acupressure or application of the acupressure device. Length of acupressure therapy varied significantly among studies due to the nature of the treated conditions. The shortest intervention (three minutes) emerged in one study focusing on pre-hospital analgesia [33] while the longest treatment was five months for cancer-related fatigue [16]. Fifty studies reported the intensity for acupressure, 24 claimed equal intensity between groups, while sham groups received only light acupressure or no stimulation in the other 26 studies.

**Table 3:** True and Sham Acupressure Protocols of the Included Studies.

Study	Practitioner	True Acupressure (TA)	Sham Acupressure (SA)	Intervention Duration	Acupressure Intensity
**Type 1—Sham Acupressure at Non-Acupoints: Manual Acupressure (19 Studies)**					
**Tang et al. 2014** [16]	Nurse Students with Acupressure Training	**Method(s)**: Manual Acupressure at True Acupoints, **Selected Acupoint(s)**: (Bilateral) Hegu (LI4), Zusanli (ST36), and Sanyinjiao (SP6)	**Method(s)**: Manual Acupressure at Non-Acupoints, **Selected Acupoint(s)**: (Bilateral) Non-Acupoints at Inner Ankle, Patella, and the First Metacarpal Head	**Total Duration**: Daily Treatment for Consecutive 5 Months, **Each Treatment**: 6 Min with 1 Min for Each Acupoint	Not Reported
**Atrian et al. 2013** [17]	Researcher with Acupressure Training	**Method(s)**: Manual Acupressure at True Acupoints, **Selected Acupoint(s)**: (Bilateral) Taichong (LR3)	**Method(s)**: Manual Acupressure at Non-Acupoints, **Selected Acupoint(s)**: (Bilateral) Non-Acupoints located 2 cm above the Distance between the Third and Fourth Toes	**Total Duration**: NR (3 Treatments in Total), **Each Treatment**: 16 Mins (Each Acupoint on Each Leg for 2 Min Pressure and 2 Min Resting, Repeated Twice)	Not Reported
**Sehhatie-Shafaie et al. 2013** [18]	Research Assistant with Acupressure Training	**Method(s)**: Manual Acupressure at True Acupoints, **Selected Acupoint(s)**: (Bilateral) Sanyinjiao (SP6), and Hegu (LI4)	**Method(s)**: Manual Acupressure at Non-Acupoints, **Selected Acupoint(s)**: (Bilateral) Non-Acupoints on the Legs and Hands (Details not Described)	**Total Duration**: Four Treatments in Total, **Each Treatment**: 20 Min	Not Reported
**Chao et al. 2013** [19]	Research Nurse with Acupressure Training	**Method(s)**: Manual Acupressure at True Acupoints, **Selected Acupoint(s)**: (Bilateral) Zusanli (ST36)	**Method(s)**: Manual Acupressure at Non-Acupoints, **Selected Acupoint(s)**: (Bilateral) Non-Acupoint located at the Tibia	**Total Duration**: 3 Treatments Per Day for 5 Days, **Each Treatment**: 5 Continuous 1-second Press followed by a 2-Second Rest, Repeated for 3 Min	Equal for Both TA and SA
**McFadden et al. 2012** [20]	Acupressure Practitioner	**Method(s)**: Manual Acupressure at True Acupoints, **Selected Acupoint(s)**: True Acupoints (Details not Described)	**Method(s)**: Manual Acupressure at Non-Acupoints, **Selected Acupoint(s)**: Non-Acupoints which cannot be Found on Established Acupressure Point Charts (Details not Described)	**Total Duration:** 40 Min (Only One Treatment)	Not Reported
**Valiee et al. 2012** [21]	Researcher with Acupressure Training	**Method(s)**: Manual Acupressure at True Acupoints, **Selected Acupoint(s)**: Yintang (or Extra-1, EX-NH3), and Shenmen (Ear)	**Method(s)**: Manual Acupressure at Non-Acupoints, **Selected Acupoint(s)**: Non-Acupoints located at the External Corner of the Left Eyebrow, and the Entrance of the Cavity of the Ear	**Total Duration**: 10 Min (Only One Treatment)	Not Reported
**Rad et al. 2012** [22]	Researcher with the Certificate of Conducting Acupressure	**Method(s)**: Manual Acupressure at True Acupoints, **Selected Acupoint(s)**: (Bilateral) Youmen (KI21)	**Method(s)**: Manual Acupressure at Non-Acupoints, **Selected Acupoint(s)**: (Unilateral) No-Acupoint (Details Not Described)	**Total Duration**: Daily Treatment for 4 Consecutive Days, **Each Treatment**: 2 Min Acupressure followed by 2 more Min Massage at the Acupoints, Repeated for 20 Min	Equal for Both TA and SA
**Chang et al. 2011** [23]	Researcher with Acupressure Training	**Method(s)**: Manual Acupressure at True Acupoints, **Selected Acupoint(s)**: Zhongji (CV3), Guanyuan (CV4), Qihai (CV6), Shenshu (BL23), Pangguangshu (BL28), Ciliao (BL32), Sanyinjiao (SP6), and Zusanli (ST36)	**Method(s)**: Manual Acupressure at Non-Acupoints, **Selected Acupoint(s)**: Non-Acupoints (Details Not Described)	**Total Duration**: 3 Treatments Per Week for 10 Weeks, **Each Treatment**: 30 Min	Unequal (Less in SA, Only Light Pressure)
**McFadden et al. 2011** [24]	Acupressure Practitioner	**Method(s)**: Manual Acupressure at True Acupoints, **Selected Acupoint(s)**: True Acupoints (Details not Described)	**Method(s)**: Manual Acupressure at Non-Acupoints, **Selected Acupoint(s)**: Non-Acupoints which cannot be Found on Established Acupressure Point Charts (Details not Described)	**Total Duration**: 2 Treatments Per week for 4 Weeks, **Each Treatment**: 40 Min	Not Reported
**Kashefi et al. 2011** [25]	Researcher with Acupressure Training	**Method(s)**: Manual Acupressure at True Acupoints, **Selected Acupoint(s)**: (Bilateral) Sanyinjiao (SP6)	**Method(s)**: Manual Acupressure at Non-Acupoints, **Selected Acupoint(s)**: (Bilateral) Non-Acupoint located at the Dorsal Side of the Leg, Away from Meridians and is not Stimulated upon the Achilles Tendon	**Total Duration**: NR (One Treatment for Each Menstrual Cycle, 2 Treatments in Total), **Each Treatment**: 6-Second Pressure followed by 2-Second Rest, Repeated for 30 Min	Equal for Both TA and SA
**Reza et al. 2010** [26]	Investigator with Acupressure Training	**Method(s)**: Manual Acupressure at True Acupoints, **Selected Acupoint(s)**: (Bilateral) Neiguan (P6), Shenmen (Hand, HT7), Shenmen (Ear), Yungchuan (KI1), Sanyinjiao (SP6), and Anmian (EX-HN22)	**Method(s)**: Manual Acupressure at Non-Acupoints, **Selected Acupoint(s)**: Non-Acupoints Located 0.5 cun Away from Meridian	**Total Duration**: 3 Treatments Per Week for 4 Weeks	Not Reported
**McFadden & Hernández 2010** [27]	Acupressure Practitioner	**Method(s)**: Manual Acupressure at True Acupoints, **Selected Acupoint(s)**: True Acupoints (Details not Described)	**Method(s)**: Manual Acupressure at Non-Acupoints, **Selected Acupoint(s)**: Non-Acupoints which cannot be Found on Established Acupressure Point Charts (Details not Described)	**Total Duration**: One Treatment Per Week for 8 Weeks, **Each Treatment**: 40 Min	Not Reported
**Maa et al. 2007** [28]	Investigator Skilled in Acupressure	**Method(s)**: Manual Acupressure at True Acupoints, **Selected Acupoint(s)**: (Bilateral) Zhongfu(LU1), Chize (LU5), Yuji (LU10), Fenglong (ST40), and Zusanli (ST36)	**Method(s)**: Manual Acupressure at Non-Acupoints, **Selected Acupoint(s)**: (Bilateral) Non-Acupoints located near Real Acupoints (where no Acupoints are Known to Exist)	**Total Duration**: Daily Treatment (at Least) for 8 Weeks, **Each Treatment**: 30 Seconds to 2 Min Acupressure for Each Acupoint (Total Duration for Each Treatment not Mentioned)	Not Reported
**Shin et al. 2007** [29]	Nurse with Acupressure Training	**Method(s)**: Manual Acupressure at True Acupoints, **Selected Acupoint(s)**: Neiguan (P6)	**Method(s)**: Manual Acupressure at Non-Acupoints, **Selected Acupoint(s)**: Non-Acupoint located at a Bony Part around the Radial Pulse at the Wrist	**Total Duration**: 3 Treatments Per Day, From the Second Day of Hospitalization to the Day before Discharge, **Each Treatment**: 10 Min (7 Seconds Acupressure Followed by 2 Seconds Rest, Repeated for 10 Min)	Not Reported
**Bertalanffy et al. 2004** [30]	Paramedic	**Method(s)**: Manual Acupressure at True Acupoints using A Hard Plastic Ball, **Selected Acupoint(s)**: (Bilateral) Korean Hand Acupressure Point located at the Middle Phalanx of the Fourth Finger (K-K9)	**Method(s)**: Manual Acupressure at Non-Acupoints using A Hard Plastic Ball, **Selected Acupoint(s)**: (Bilateral) Non-Acupoint located at the Middle Phalanx of the Second Finger	**Total Duration**: From the Time of Randomization to Arrival at the Hospital	Not Reported
**Chen et al. 2003** [31]	Research Assistant with Acupressure Training	**Method(s)**: Manual Acupressure at True Acupoints, **Selected Acupoint(s)**: Neiguan (P6), Zusanli (ST36), and Sanyinjiao (SP6)	**Method(s)**: Manual Acupressure at Non-Acupoints, **Selected Acupoint(s)**: Non-Acupoints located at the Tibia or Radial Bone Surface Approximately 3–4 cm from the Selected True Acupoints	**Total Duration**: 4 Days in Hospital and Self-Acupressure at Home in the Following Days (Details Not Described), **Each Treatment**: 9 Min (3 Min for Each Acupoint)	Not Reported
**Tsay & Chen 2003** [32]	Investigator and Research Assistant with Acupressure Training	**Method(s)**: Manual Acupressure at True Acupoints, **Selected Acupoint(s)**: (Bilateral) Shenmen (Hand, HT7), Shenmen (Ear), Yungchuan (KI1)	**Method(s)**: Manual Acupressure at Non-Acupoints, **Selected Acupoint(s)**: (Bilateral) Non-Acupoints located 1 cm away from Meridian	**Total Duration**: 3 Treatments Per Week for 4 Weeks, **Each Treatment**: 14 Min	Not Reported
**Kober et al. 2002** [33]	Paramedic with Acupressure Training	**Method(s)**: Manual Acupressure at True Acupoints, **Selected Acupoint(s)**: Zhongchong (PC9), Neiguan (P6), Kunlun (BL60), Hegu (LI4), and Baihui (GV20)	**Method(s)**: Manual Acupressure at Non-Acupoints, **Selected Acupoint(s)**: Non-Acupoints located at the Middle of Dorsal Wrist, Middle of Clavicle, Middle of Patella, Lateral Metacarpophalangeal Junction of the Second Finger, and Middle of Lateral Malleolus	**Total Duration**: 3 Min	Not Reported
**Belluomini et al. 1994** [34]	Not Reported	**Method(s)**: Manual Acupressure at True Acupoints, **Selected Acupoint(s)**: Neiguan (P6)	**Method(s)**: Manual Acupressure at Non-Acupoints, **Selected Acupoint(s)**: Non-Acupoint located at the Palmar Surface of the Hand, Proximal to the Head of the Fifth Metacarpal Joint	**Total Duration**: 4 Times Per Day for Consecutive 7 Days, **Each Treatment**: 10 Min	Not Reported
**Type 2—Sham Acupressure at Non-Acupoints: Acupressure Bands or Other Devices (21 Studies)**					
**Adib-Hajbaghery & Etri, 2013** [35]	Researcher with Acupressure Training	**Method(s)**: Acupressure at True Acupoints using Acupressure Band (PsiBand), **Selected Acupoint(s)**: (Unilateral) Lanwei (EX-LE7)	**Method(s)**: Acupressure at Non-Acupoints using Acupressure Band (PsiBand), **Selected Acupoint(s)**: (Unilateral) Non-Acupoint located at the Opposite Site of the Lanwei (EX-LE7) Point	**Total Duration**: 7 Hours (Loosened for 10 Min every 2 Hours and then Tightened)	Unequal (Less in SA)
**Alessandrini et al. 2012** [36]	Not Reported	**Method(s)**: Acupressure at True Acupoints using Acupressure Band (Sea-Band), **Selected Acupoint(s)**: (Bilateral) Neiguan (P6)	**Method(s)**: Acupressure at Non-Acupoints using Acupressure Band (Sea-Band), **Selected Acupoint(s)**: (Bilateral) Non-Acupoint located at the Dorsal Part of the Carpus	**Total Duration**: 30 Min	Equal for Both TA and SA
**Soltani et al. 2011** [37]	Not Reported	**Method(s)**: Using Acupressure Wrist Band at True Acupoints, **Selected Acupoint(s)**: (Bilateral) Neiguan (P6)	**Method(s)**: Using Acupressure Wrist Band at Non-Acupoints, **Selected Acupoint(s)**: (Bilateral) Non-Acupoint located at the Posterior Surface of Both Forearms	**Total Duration**: Applied 30 Min before Induction of Anesthesia and Removed 6 Hours Later	Equal for Both TA and SA
**Bao et al. 2011** [38]	Acupressure Operator	**Method(s)**: Acupressure at True Acupoints using Magnetic Acupressure Suction Cups + Standard Local Analgesics, **Selected Acupoint(s)**: (Bilateral) Hegu (LI4)	**Method(s)**: Acupressure at Non-Acupoints using Magnetic Acupressure Suction Cups, **Selected Acupoint(s)**: (Bilateral) Non-Acupoint located at the Proximal Dorsum of the Fourth Interosseus Space of the Hand	**Total Duration**: The Entire BMAB Procedure	Equal for Both TA and SA
**Majholm & Møller 2011** [39]	Nursing Assistant	**Method(s)**: Acupressure at True Acupoints using Acupressure Band (Vital-Band), **Selected Acupoint(s)**: (Unilateral) Neiguan (P6)	**Method(s):** Acupressure at Non-Acupoints using Acupressure Band (Vital-Band), **Selected Acupoint(s)**: (Unilateral) Non-Acupoint located at the Dorsum of the Forearm	**Total Duration**: Applied just before Induction of Anesthesia and Kept for 24 Hours after Surgery	Equal for Both TA and SA
**Sinha et al. 2011** [40]	Not Reported	**Method(s)**: Acupressure at True Acupoints using Acupressure Band (Pressure Right^TM^), **Selected Acupoint(s)**: (Bilateral) Neiguan (P6)	**Method(s)**: Acupressure at Non-Acupoints using Placebo Acupressure Band, **Selected Acupoint(s)**: (Bilateral) Non-Acupoint located at A Distance of 3 of the Parturient’s Finger-Breadths from the Proximal Palmar Crease	**Total Duration**: From the Time of Randomization to 2 Hours after Vaginal Delivery or the Time of A Decision of Caesarean Delivery	Unequal (No Pressure was Applied for SA)
**Wang et al. 2008** [41]	Acupuncturist	**Method(s)**: Acupressure at True Acupoints using Acupressure Beads, **Selected Acupoint(s)**: Yintang (or Extra-1, EX-NH3)	**Method(s)**: Acupressure at Non-Acupoints using Acupressure Beads, **Selected Acupoint(s)**: Non-Acupoint (Above the Lateral Boarder of the Left Eyebrow)	**Total Duration**: Entire Anesthesia Period (Applied before Enter the Operation Room and Removed at the Completion of the Procedure)	Equal for Both TA and SA
**Turgut et al. 2007** [42]	Anesthesiologist	**Method(s)**: Acupressure at True Acupoints using Acupressure Band (Sea-Band), **Selected Acupoint(s)**: (Bilateral) Neiguan (P6)	**Method(s)**: Acupressure at Non-Acupoints using Acupressure Band (Sea-Band), **Selected Acupoint(s)**: (Bilateral) Non-Acupoint located at the Dorsal Surface of the Forearm	**Total Duration**: Applied 30 Min before Anesthesia and Kept for 24 Hours after Surgery	Equal for Both TA and SA
**Heazell et al. 2006** [43]	Not Reported	**Method(s)**: Acupressure at True Acupoints using Acupressure Band (Sea-Band), **Selected Acupoint(s)**: (Bilateral) Neiguan (P6)	**Method(s)**: Acupressure at Non-Acupoints using Acupressure Band (Sea-Band), **Selected Acupoint(s)**: (Bilateral) Non-Acupoint located at the Dorsal Aspect of the Forearm	**Total Duration**: Not Reported (8 Hours Per Day)	Equal for Both TA and SA
**Wang et al. 2005** [44]	Acupuncturist	**Method(s)**: Acupressure at True Acupoints using Acupressure Beads, **Selected Acupoint(s)**: Yintang (or Extra-1, EX-NH3)	**Method(s)**: Acupressure at Non-Acupoints using Acupressure Beads, **Selected Acupoint(s)**: Non-Acupoint (Above the Lateral Boarder of the Left Eyebrow)	**Total Duration**: More than 20 Min	Equal for Both TA and SA
**Alkaissi et al. 2005** [45]	Not Reported	**Method(s)**: Acupressure at True Acupoints using Acupressure Band (Sea-Band), **Selected Acupoint(s)**: (Bilateral) Neiguan (P6)	**Method(s)**: Acupressure at Non-Acupoints using Acupressure Band (Sea-Band), **Selected Acupoint(s)**: (Bilateral) Non-Acupoint located at the Dorsal Side of both Forearms, 4 Fingers Breadth Proximal to the Proximal Flexor Palmar Crease	**Total Duration**: Not Reported	Equal for Both TA and SA
**Samad et al. 2003** [46]	Investigator	**Method(s)**: Acupressure at True Acupoints using Wrist-Band with Plastic Bead, **Selected Acupoint(s)**: (Unilateral) Neiguan (P6)	**Method(s)**: Acupressure at Non-Acupoints using Wrist-Band with Plastic Bead, **Selected Acupoint(s)**: (Unilateral) Non-Acupoint located at the Dorsum of the Right Forearm away from P6 Acupoint	**Total Duration**: Applied 30 Min before Induction of Anesthesia, and Kept for 6 Hours after Surgery	Equal for Both TA and SA
**Alkaissi et al. 2002** [47]	Nurse	**Method(s)**: Acupressure at True Acupoints using Acupressure Band (Sea-Band), **Selected Acupoint(s)**: (Bilateral) Neiguan (P6)	**Method(s)**: Acupressure at Non-Acupoints using Acupressure Band (Sea-Band), **Selected Acupoint(s)**: (Bilateral) Non-Acupoint located at the Dorsal Site of the Forearm, 4 Fingers Breadth Proximal to the Flexor Palmar Crease	**Total Duration**: 24 Hours	Equal for Both TA and SA
**Agarwal et al. 2000** [48]	Individual who was Trained in the Application of Acupressure Band	**Method(s)**: Acupressure at True Acupoints using Acupressure Bands, **Selected Acupoint(s)**: (Bilateral) Neiguan (P6)	**Method(s)**: Acupressure at Non-Acupoints using Acupressure Bands, **Selected Acupoint(s)**: (Bilateral) Non-Acupoint located at the Posterior Surface of the Wrist	**Total Duration**: Applied 30 Min before Induction of Anesthesia, and Removed 6 Hours after Surgery	Equal for Both TA and SA
**Harmon et al. 2000** [49]	Anesthetist	**Method(s)**: Acupressure at True Acupoints using Acupressure Band (Sea-Band), **Selected Acupoint(s)**: (Unilateral) Neiguan (P6)	**Method(s)**: Acupressure at Non-Acupoints using Acupressure Band (Sea-Band), **Selected Acupoint(s)**: (Unilateral) Non-Acupoint located at the Dorsal Site of the Right Forearm	**Total Duration**: Applied 5 Min before Induction of Anesthesia, and Removed 6 Hours after Discharge to the Ward	Equal for Both TA and SA
**Harmon et al. 1999** [50]	Not Reported	**Method(s)**: Acupressure at True Acupoints using Acupressure Band (Sea-Band), **Selected Acupoint(s)**: (Unilateral) Neiguan (P6)	**Method(s)**: Acupressure at Non-Acupoints using Acupressure Band (Sea-Band), **Selected Acupoint(s)**: (Unilateral) Non-Acupoint (Details Not Described)	**Total Duration**: Applied immediately before Induction of Anesthesia, and Removed 20 Min after Induction of Anesthesia	Equal for Both TA and SA
**Alkaissi et al. 1999** [51]	Researcher	**Method(s)**: Acupressure at True Acupoints using Acupressure Band (Sea-Band), **Selected Acupoint(s)**: (Bilateral) Neiguan (P6)	**Method(s)**: Acupressure at Non-Acupoints using Acupressure Band (Sea-Band), **Selected Acupoint(s)**: (Bilateral) Non-Acupoint located at the Dorsal Site of Both Forearms, 4 Fingers Breadth Proximal to the Proximal Flexor Palmar Crease	**Total Duration**: Applied just before Surgery, and Removed after Discharge to Home (Details not Described)	Equal for Both Groups
**Fan et al. 1997** [52]	Individual who was Trained in the Application of Acupressure Band	**Method(s)**: Acupressure at True Acupoints using Acupressure Band (AcuBand), **Selected Acupoint(s)**: (Bilateral) Neiguan (P6)	**Method(s)**: Acupressure at Non-Acupoints using Acupressure Band (AcuBand), **Selected Acupoint(s)**: (Bilateral) Non-Acupoint located at the Dorsum of Both Wrists	**Total Duration**: Applied before Induction of Anesthesia, and Removed 6 Hours after Surgery	Unequal (AcuBands were Tied Loosely in SA)
**O’Brien et al. 1996** [53]	Not Reported	**Method(s)**: Acupressure at True Acupoints using Acupressure Band (Sea-Band), **Selected Acupoint(s)**: (Bilateral) Neiguan (P6)	**Method(s)**: Acupressure at Non-Acupoints using Acupressure Band (Sea-Band), **Selected Acupoint(s)**: (Bilateral) Non-Acupoint located at the Radius of Both Forearms	**Total Duration**: 3 Days (From the Morning of Study Day 3 to The Morning of Study Day 6)	Equal for Both TA and SA
**Felhendler & Lisander 1996** [54]	Researcher	**Method(s)**: Acupressure at True Acupoints using A Dentist’s Tool (A 15-cm Handle with A Ball at the End), **Selected Acupoint(s)**: (Unilateral) Chengqi (ST1), Lidui (ST45), Yinbai (SP1), Dabao (SP21), Gongsun (SP4), Jingming (BL1), Zhiyin (BL67), Yungchuan (KI1), Youmen (KI27), Dazhong (KI4), Ligou (LR5), Tongziliao (GB1), Zuqiaoyin (GB44), Dadun (LR1), and Qimen (LR14)	**Method(s)**: Acupressure at Non-Acupoints using A Dentist’s Tool (A 15-cm Handle with A Ball at the End), **Selected Acupoint(s)**: (Unilateral) Non-Acupoints Situated 2 cm from the Nearest True Acupoints	**Total Duration**: 30 Min after Patients Awoke from Anesthesia	Unequal (Only Light Pressure for SA)
**Bayreuther et al. 1994** [55]	Not Reported	**Method(s)**: Acupressure at True Acupoints using Acupressure Band (Sea-Band), **Selected Acupoint(s)**: Neiguan (P6)	**Method(s)**: Acupressure at Non-Acupoints using Acupressure Band (Sea-Band), **Selected Acupoint(s)**: Non-Acupoint located Above or Below the Elbow	**Total Duration**: 7 Days	Equal for Both TA and SA
**Type 3—Pseudo-Intervention at the Same Acupoints as Intervention Group: Manual Light-Touch without Acupressure (7 Studies)**					
**Hamidzadeh et al. 2012** [56]	Researcher with Acupressure Training	**Method(s)**: Manual Acupressure at True Acupoints, **Selected Acupoint(s)**: (Bilateral) Hegu (LI4)	**Method(s)**: Light Touch (without Acupressure) on the Same Acupoints, **Selected Acupoint(s)**: (Bilateral) Hegu (LI4)	**Total Duration**: 20 Min (5 Pressure Per Min)	No Pressure was Applied for SA
**Mirbagher-Ajorpaz et al. 2011** [57]	Researcher with Acupressure Training	**Method(s)**: Manual Acupressure at True Acupoints, **Selected Acupoint(s)**: Sanyinjiao (SP6)	**Method(s)**: Light Touch (without Acupressure) on the Same Acupoints, **Selected Acupoint(s)**: Sanyinjiao (SP6)	**Total Duration**: 20 Min (10 Second-Cycle repeated 120 Times)	No Pressure was Applied for SA
**Sun et al. 2010** [58]	Research Assistant with Acupressure Training	**Method(s)**: Manual Acupressure at True Acupoints, **Selected Acupoint(s)**: (Bilateral) Shenmen (HT7)	**Method(s)**: Light Touch (without Acupressure) on the Same Acupoints, **Selected Acupoint(s)**: (Bilateral) Shenmen (HT7)	**Total Duration**: Daily Treatment for Consecutive 5 Weeks, **Each Treatment**: 5-Second Pressure Followed by 1-Second Rest, Repeated for 5 Min	No Pressure was Applied for SA
**Kashanian & Shahali 2010** [59]	Investigator	**Method(s)**: Manual Acupressure at True Acupoints, **Selected Acupoint(s)**: Sanyinjiao (SP6)	**Method(s)**: Light Touch (without Acupressure) on the Same Acupoints, **Selected Acupoint(s)**: Sanyinjiao (SP6)	**Total Duration**: 30 Min	No Pressure was Applied for SA
**Hjelmstedt et al. 2010** [60]	A Person with Acupressure Training	**Method(s)**: Manual Acupressure at True Acupoints, **Selected Acupoint(s)**: (Bilateral) Sanyinjiao (SP6)	**Method(s)**: Light Touch (without Acupressure) on the Same Acupoints, **Selected Acupoint(s)**: (Bilateral) Sanyinjiao (SP6)	**Total Duration**: 30 Min	No Pressure was Applied for SA
**Hsu et al. 2006** [61]	Researcher with TCM Training	**Method(s)**: Manual Acupressure at True Acupoints, **Selected Acupoint(s)**: (Bilateral) Shenmen (HT7)	**Method(s)**: Light Touch (without Acupressure) on the Same Acupoints, **Selected Acupoint(s)**: (Bilateral) Shenmen (HT7)	**Total Duration**: Daily Treatment (5 Min) for Consecutive 5 Weeks	No Pressure was Applied for SA
**Lee et al. 2004** [62]	Research Intervener	**Method(s)**: Manual Acupressure at True Acupoints, **Selected Acupoint(s)**: (Bilateral) Sanyinjiao (SP6)	**Method(s)**: Light Touch (without Acupressure) on the Same Acupoints, **Selected Acupoint(s)**: (Bilateral) Sanyinjiao (SP6)	**Total Duration**: 30 Min During Each Uterine Contraction	No Pressure was Applied for SA
**Type 4—Pseudo-Intervention at the Same Acupoints as Intervention Group: Placebo Acupressure Devices (14 Studies)**					
**Nilsson et al. 2015** [63]	Nurse Anesthetist	**Method(s)**: Acupressure at True Acupoints using Acupressure Band (Sea-Band), **Selected Acupoint(s)**: (Unilateral) Neiguan (P6)	**Method(s)**: Sham Acupressure at the Same Acupoints using A Placebo Acupressure Band without the Plastic Button, **Selected Acupoint(s)**: (Unilateral) Neiguan (P6)	**Total Duration**: 2 Days	No Pressure was Applied for SA
**Molassiotis et al. 2013** [64]	Not Reported	**Method(s)**: Acupressure at True Acupoints using Acupressure Band (Sea-Band), **Selected Acupoint(s)**: (Bilateral) Neiguan (P6)	**Method(s)**: Sham Acupressure at the Same Acupoints using A Placebo Acupressure Band with the Plastic Button located at the Exterior of the Wrist Band, **Selected Acupoint(s)**: (Bilateral) Neiguan (P6)	**Total Duration**: 7 Days	No Pressure was Applied for SA
**Noroozinia et al. 2013** [65]	Not Reported	**Method(s)**: Acupressure at True Acupoints using Acupressure Band, **Selected Acupoint(s)**: Neiguan (P6)	**Method(s)**: Sham Acupressure at the Same Acupoints using A Placebo Acupressure Band without the Pressure Button, **Selected Acupoint(s)**: Neiguan (P6)	**Total Duration**: 30 Min Prior to Spinal Anesthesia	No Pressure was Applied for SA
**Soltanzadeh et al. 2012** [66]	Not Reported	**Method(s)**: Acupressure at True Acupoints using Acupressure Band, **Selected Acupoint(s)**: (Bilateral) Korean Hand Acupressure Point (K-K9) located at the Middle Phalanx of the Fourth Finger on Both Hands	**Method(s)**: Sham Acupressure at the Same Acupoints using A Dummy Band (Details not Described), **Selected Acupoint(s)**: (Bilateral) Korean Hand Acupressure Point (K-K9) located at the Middle Phalanx of the Fourth Finger on Both Hands	**Total Duration**: Applied 15 Min Prior to Induction of Anesthesia and Kept for 24 Hours	Not Reported
**White et al. 2012** [67]	Investigator	**Method(s)**: Acupressure at True Acupoints using Acupressure Strip, **Selected Acupoint(s)**: (Bilateral) Neiguan (P6)	**Method(s)**: Sham Acupressure at the Same Acupoints using A Placebo Acupressure Strip without the Pressure Button, **Selected Acupoint(s)**: (Bilateral) Neiguan (P6)	**Total Duration**: Applied 30 to 60 Min before Induction of Anesthesia and Kept for 72 Hours after Surgery	No Pressure was Applied for SA
**Ho et al. 2006** [68]	Acupuncturist	**Method(s)**: Acupressure at True Acupoints using Acupressure Band (Sea-Band), **Selected Acupoint(s)**: (Bilateral) Neiguan (P6)	**Method(s)**: Sham Acupressure at the Same Acupoints using A Placebo Acupressure Band with A Blunted Plastic Button, **Selected Acupoint(s)**: (Bilateral) Neiguan (P6)	**Total Duration**: Applied More than 30 Min before Induction of Anesthesia and Removed after Arrival in the Post-Anesthesia Care Unit	No Pressure was Applied for SA
**Klein et al. 2004** [69]	Not Reported	**Method(s)**: Acupressure at True Acupoints using Acupressure Band (Sea-Band), **Selected Acupoint(s)**: (Bilateral) Neiguan (P6)	**Method(s)**: Sham Acupressure at the Same Acupoints using A Placebo Acupressure Band without the Plastic Button, **Selected Acupoint(s)**: (Bilateral) Neiguan (P6)	**Total Duration**: Applied before Induction of Anesthesia and Removed 24 Hours after Extubation	No Pressure was Applied for SA
**Schultz et al. 2003** [70]	Preoperative Nurse	**Method(s)**: Acupressure Band (Sea-Band) at True Acupoints + Droperidol, **Selected Acupoint(s)**: Neiguan (P6)	**Method(s)**: Placebo Band (with A Flat Button) at the Same Acupoints, **Selected Acupoint(s)**: Neiguan (P6)	**Total Duration**: Not Reported	No Pressure was Applied for SA
**Dent et al. 2003** [71]	Nurse who was Trained in Accurate Location of Targeted Acupoints	**Method(s)**: Acupressure at True Acupoints using Acupressure Band (Sea-Band), **Selected Acupoint(s)**: (Bilateral) Neiguan (P6)	**Method(s)**: Sham Acupressure at the Same Acupoints using A Placebo Acupressure Band without the Plastic Button, **Selected Acupoint(s)**: (Bilateral) Neiguan (P6)	**Total Duration**: 24 Hours	No Pressure was Applied for SA
**Norheim et al. 2001** [72]	Study Assistant	**Method(s)**: Acupressure at True Acupoints using Acupressure Band (Sea-Band), **Selected Acupoint(s)**: (Bilateral) Neiguan (P6)	**Method(s)**: Sham Acupressure at the Same Acupoints using A Placebo Acupressure Band without the Plastic Button, **Selected Acupoint(s)**: (Bilateral) Neiguan (P6)	**Total Duration**: 4 Days	No Pressure was Applied for SA
**Steele et al. 2001** [73]	Nurse who was Trained in the Application of Acupressure Band	**Method(s)**: Acupressure at True Acupoints using Acupressure Band (Sea-Band), **Selected Acupoint(s)**: (Bilateral) Neiguan (P6)	**Method(s)**: Sham Acupressure at the Same Acupoints using A Placebo Acupressure Band without the Plastic Button, **Selected Acupoint(s)**: (Bilateral) Neiguan (P6)	**Total Duration**: 4 Days	No Pressure was Applied for SA
**Woods 1999** [74]	Anesthesia Provider	**Method(s)**: Acupressure at True Acupoints using Acupressure Band (Sea-Band), **Selected Acupoint(s)**: (Bilateral) Neiguan (P6)	**Method(s)**: Sham Acupressure at the Same Acupoints using A Placebo Acupressure Band without the Plastic Button, **Selected Acupoint(s)**: (Bilateral) Neiguan (P6)	**Total Duration**: Applied at Least 15 Min before Induction of Anesthesia and Kept for 24 Hours after Surgery	No Pressure was Applied for SA
**Duggal et al. 1998** [75]	Nurse	**Method(s)**: Acupressure at True Acupoints using Acupressure Band (Sea-Band), **Selected Acupoint(s)**: (Bilateral) Neiguan (P6)	**Method(s)**: Sham Acupressure at the Same Acupoints using A Placebo Acupressure Band without the Plastic Button, **Selected Acupoint(s)**: (Bilateral) Neiguan (P6)	**Total Duration**: At Least 10 Hours	No Pressure was Applied for SA
**Lewis et al. 1991** [76]	Investigator	**Method(s)**: Acupressure at True Acupoints using Acupressure Band (Sea-Band), **Selected Acupoint(s)**: (Bilateral) Neiguan (P6)	**Method(s)**: Sham Acupressure at the Same Acupoints using A Placebo Acupressure Band without the Stud, **Selected Acupoint(s)**: (Bilateral) Neiguan (P6)	**Total Duration**: Applied 1 Hour before Operation and Kept until Discharge from Hospital on the same Day	No Pressure was Applied for SA
**Type 5—Acupressure at Non-Therapeutic Acupoints (Irrelevant Acupoints): Manual Acupressure (4 Studies)**					
**Suh 2012** [77]	Researcher and Research Assistant	**Method(s)**: Acupressure at Therapeutic Acupoints using Acupressure Band (Sea-Band), **Selected Acupoint(s)**: (Bilateral) Neiguan (P6)	**Method(s)**: Manual Acupressure at Non-Therapeutic (Irrelevant) Acupoints, **Selected Acupoint(s)**: (Bilateral) Houxi (SI3)	**Total Duration**: 5 Days	Unequal (Continuously Automatic Pressure for TA and Manual Acupressure for SA)
**Lang et al. 2007** [78]	Paramedic with Acupressure Training	**Method(s)**: Manual Acupressure at Therapeutic Acupoints, **Selected Acupoint(s)**: Baihui (GV20) and Hegu (LI4, Unilateral)	**Method(s)**: Manual Acupressure at Non-Therapeutic (Irrelevant) Acupoints, **Selected Acupoint(s)**: Geshu (BL17) and Jianliao (TE14, Unilateral)	**Total Duration**: Not Reported (3 Min Acupressure for Each Acupoint)	Equal for Both TA and SA
**Wu et al. 2007** [79]**Wu et al. 2004** [80]	Investigator with Acupressure Training	**Method(s)**: Manual Acupressure at Therapeutic Acupoints, **Selected Acupoint(s)**: Dazhui (GV14), Tiantu (CV22), Feishu (B13, Bilateral), Shenshu (B23, Bilateral), and Yuji (L10, Unilateral)	**Method(s)**: Manual Acupressure at Non-Therapeutic (Irrelevant) Acupoints, **Selected Acupoint(s)**: Shangqiu (SP5), Taibai (SP3), and Dadun (LR1)	**Total Duration**: 5 Treatments Per Week for Consecutive 4 Weeks, **Each Treatment**: 16 Min	Equal for Both TA and SA
**Type 6—Acupressure at Non-Therapeutic Acupoints (Irrelevant Acupoints): Acupressure Bands or Other Devices (1 Study)**					
**Nordio & Romanelli 2008** [81]	Not Reported	**Method(s)**: Acupressure at Therapeutic Acupoints using Wrist Acupressure Device, **Selected Acupoint(s)**: (Bilateral): Shenmen (HT7)	**Method(s)**: Acupressure at Non-Therapeutic (Irrelevant) Acupoints using Wrist Acupressure Device, **Selected Acupoint(s)**: (Bilateral): Acupoint Known as not to Interfere with Insomnia and/or Anxiety (Details not Described)	**Total Duration**: 20 Days	Equal for Both TA and SA

For the types of acupressure, 61 articles employed Chinese acupuncture theory to guide the acupressure protocol, and three adopted the Japanese Jin Shin acupressure [20, 24, 27], while the other two used Korean hand acupressure [30,66]. Bilateral acupoints were stimulated in both groups in 38 studies, and eight trials only employed unilateral stimulation, while in the other 20 studies, both bilateral and unilateral acupoints were used or such information were not reported. The number of selected acupoints ranged from one to 15 in the true treatment groups, and the majority (50 studies) only applied one acupoint (bilateral or unilateral) for acupressure. In one study on postoperative pain [54], 15 acupoints were chosen for stimulation. Selection of the acupoints was based on the targeted health issue, and *neiguan* (P6) was frequently used for controlling nausea and/or vomiting, *sanyinjiao* (SP6) and *hegu* (LI4) were used for treating various types of pain such as peri-operative pain, primary dysmenorrheal and labor pain, *shenmen* (HT7) was mainly used for sleep disorders, and *yintang* (EX-NH3) was used for anxiety relief.

### Description of Sham Acupressure Procedures

Sham acupressure protocols are also summarized in **Table 3**. Six types of sham acupressure were identified. Non-acupoint was used in 40 studies, of which, 19 manually stimulated the non-acupoint (type 1). For studies with type 1 sham mode, 12 described the definite location of the selected non-acupoints and most of them were near the real acupoints used in the true intervention groups. More specifically, three studies [25, 26, 32] indicated that the non-acupoints should be away from the nearby meridians, with 0.5 cun (a traditional Chinese term that translates to "anatomical inch") in one study [26], and one cm in another [32]. In addition, there was one study [31] stating that the non-acupoints should be three to four cm away from the targeted active acupoints. Total duration of treatment as well as duration of each session were similar between groups. In terms of the acupressure intensity, three studies [19, 22, 25] applied equal pressure between groups, and one [23] only exerted light pressure on non-acupoints, while this information was not reported in the other 15 articles.

Twenty-one studies used acupressure devices at the non-acupoints (type 2). Both true and sham study arms utilized the same equipment. Wrist bands such as sea-bands or vital-bands, etc. were adopted in 16 studies and these tools usually possess a plastic/metal button located at the interior surface which can exert constant pressure at the targeted acupoint. However, in one study [40], the button was replaced by a backing square in the sham group. In two trials where the non-acupoints located at the forehead, adhesive beads were applied [41, 44]. Special band was also adopted on the non-acupoint located below the knee in one study [35], and magnetic suction cup was utilized in another trial to stimulate the non-acupoints on both hands [38]. Particularly, a dentist’s tool was employed for acupressure in one trial which selected more than ten non-acupoints [54]. Twenty studies described the definite locations of the non-acupoints. Most of the studies with type 2 design adopted equal acupressure intensity between groups, while it was unequal in four trials [35, 40, 52, 54] with sham groups receiving lower-intensity pressure or no stimulation at all.

In 21 articles, the sham study arms employed the same acupoints as in the true acupressure arms. Manual light touch was applied in seven trials without any pressure (type 3) whereas the other 14 used a placebo device but also no pressure was exerted (type 4). The intervention protocols were all similar between groups except for the acupressure intensity. For studies utilizing type 4 sham procedures, pseudo devices used in the control groups were identical from those applied in true acupressure with the only exception that the pressure button was missing, or was replaced by a blunted/flat one, or was located at the exterior of the equipment. One study stated that a dummy band was applied but no detailed information was given [66].

Non-therapeutic acupoints were chosen as sham intervention in five trials. Of which, four utilized manual acupressure (type 5) [77–80] and one employed a wrist acupressure device (type 6) [81]. In studies with type 5 sham method, the intervention protocol was similar between groups in 3 studies [78–80], but in another trial [77], the acupressure intensity was unequal between groups with the true intervention group applying a wrist band to create constant pressure while the sham comparison received manual acupressure with discontinuous stimulation. The only one article with type 6 sham procedure focused on insomnia [81]. Unfortunately, this study failed to specify the selected sham acupoints but only stated that the irrelevant acupoints were “known as not to interfere with” the treated condition [81].

### Therapeutic Effects of Acupressure

Therapeutic outcomes of acupressure are summarized in **Tables 4** and **5**. Meanwhile, results of the responder rate ratio calculation are presented in **Table 6**.

**Table 4:** Summary of the Therapeutic Outcomes based on Different Sham Acupressure Types.

		Results of Therapeutic Effects			
Types of Sham Control	No. of Studies	TAG is Significantly Superior to SAG^1^	TAG is Superior to SAG^2^	No Difference between TAG and SAG^3^	SAG is Superior to TAG^4^
**Type 1**	18^#^	13 (72.2%)	1 (5.6%)	4 (22.2%)	0 (0%)
**Type 2**	20^##^	11 (55.0%)	3 (15.0%)	6 (30.0%)	0 (0%)
**Type 3**	7	7 (100.0%)	0 (0%)	0 (0%)	0 (0%)
**Type 4**	14	5 (35.7%)	4 (28.6%)	3 (21.4%)	2 (14.3%)
**Type 5**	4	4 (100.0%)	0 (0%)	0 (0%)	0 (0%)
**Type 6**	1	1 (100.0%)	0 (0%)	0 (0%)	0 (0%)
**Total**	64	41 (64.1%)	8 (12.5%)	13 (20.3%)	2 (3.1%)

**Type 1**: Sham acupressure at non-acupoints by manual pressure; **Type 2**: Sham acupressure at non-acupoints by employing acupressure devices; **Type 3**: Pseudo-intervention at the same acupoints as true treatment arm by manual light-touch; **Type 4**: Pseudo-intervention at the same acupoints as true treatment arm by using placebo devices; **Type 5**: Manual acupressure at non-therapeutic (irrelevant) acupoints; **Type 6**: Sham acupressure at non-therapeutic (irrelevant) acupoints by adopting acupressure devices

**1:** For studies reporting multiple main outcomes, at least one “++” were identified

**2**: For studies reporting multiple main outcomes, no “++” but at least one “+” were identified

**3**: “0” for all main outcome(s)

**4**: “−” or “− −”for all main outcome(s)

**#**: Sham acupressure control type 1 was applied in 19 studies, but comparison between groups was not performed (or not clearly reported) in one study

##: Sham acupressure control type 2 was applied in 21 studies, but comparison between groups was not performed (or not clearly reported) in one study

**Table 5:** Summary of the Therapeutic Outcomes based on Different Health Problems and Treatment Durations.

		Results of Therapeutic Effects			
Subgroups	No. of Studies	TAG is Significantly Superior to SAG^1^	TAG is Superior to SAG^2^	No Difference between TAG and SAG^3^	SAG is Superior to TAG^4^
**Health Problem**					
Peri-operative Nausea and Vomiting ^#^	21	8	5	6	2
Nausea and Vomiting in Pregnancy	9	7	0	2	0
Labor Pain	5	5	0	0	0
Sleep Disturbances	5	4	0	1	0
Perioperative/Prehospital Pain	4^##^	3	0	1	0
Anxiety	3	3	0	0	0
Symptoms in Patients with Respiratory Disorders	3	2	0	1	0
Motion Sickness	2^###^	1	1	0	0
Primary Dysmenorrhea	2	1	0	1	0
Chemotherapy-induced Nausea and Vomiting	2	1	0	1	0
Postoperative Gastrointestinal Function	2	2	0	0	0
Cancer-related Fatigue	1	0	1	0	0
Stress Reduction	1	0	0	1	0
Urodynamic Stress Incontinence	1	1	0	0	0
Traumatic Brain Injury	1	1	0	0	0
Women’s General Health	1	1	0	0	0
Cardiovascular Function in Stroke Survivors	1	1	0	0	0
Post MI-related Nausea and Vomiting	1	0	1	0	0
**Treatment Duration**					
Less than 1 Hour	12^####^	10	0	2	0
More than 1 Hour but Less than 1 Day	13	7	3	3	0
More than 1 Day but Less than 1 Week	16	8	3	4	1
More than 1 Week	16	12	1	3	0

**1:** For studies reporting multiple main outcomes, at least one “++” were identified

**2**: For studies reporting multiple main outcomes, no “++” but at least one “+” were identified

**3**: “0” for all main outcome(s)

**4**: “−” or “− −”for all main outcome(s); MI: Myocardial infarction

# Twenty studies for postoperative nausea and vomiting and one study for intra-operative nausea and vomiting

## Perioperative/prehospital pain was observed in 5 studies, but comparison between groups was not performed (or not clearly reported) in 1 study

### Motion sickness was observed in 3 studies, but comparison between groups was not performed (or not clearly reported) in 2 studies

#### Acupressure durations were less than 1 hour in 14 studies, but comparison between groups was not performed (or not clearly reported) in 2 studies

**Table 6:** Results of Response Rates for Studies Presenting Dichotomous Data.

Study	Outcome^#^	Definition of Response	TAG (n/N)	SAG (n/N)	Relative Risk 95% CI
**Type 1** Sham Acupressure at Non-Acupoints: Manual Acupressure					
Bertalanffy et al. 2004	Sympathetic Activity	Peripheral Vasodilation	46/50	2/50	23.00 [5.90, 89.64]
Kober et al. 2002	Pre-Hospital Anxiety	Anxiety Reduction	13/19	10/20	1.37 [0.80, 2.33]
**Type 2** Sham Acupressure at Non-Acupoints: Acupressure Bands or Other Devices					
Adib-Hajbaghery & Etri 2013	Postoperative Nausea	Absence of Nausea	17/35	18/35	0.94 [0.59, 1.51]
Adib-Hajbaghery & Etri 2013	Postoperative Vomiting	Absence of Vomiting	19/35	20/35	0.95 [0.63, 1.44]
Alessandrini et al. 2012	Neurovegetative Symptom	Symptom Improvement	87/102	11/102	7.91 [4.50, 13.90]
Soltani et al. 2011	Postoperative Nausea	Absence of Nausea	44/50	31/50	1.42 [1.12, 1.80]
Soltani et al. 2011	Postoperative Vomiting	Absence of Vomiting	40/50	27/50	1.48 [1.11, 1.98]
Bao et al. 2011	Pain Intensity	Less Severity Pain	36/37	32/40	1.22 [1.03, 1.43]
Majholm & Møller 2011	Postoperative Nausea	Absence of Nausea	37/57	29/51	1.14 [0.84, 1.55]
Majholm & Møller 2011	Postoperative Vomiting	Absence of Vomiting	43/58	38/52	1.01 [0.81, 1.27]
Sinha et al. 2011	Nausea and Vomiting in Delivery	Absence of Nausea and/or Vomiting	82/170	87/170	0.94 [0.76, 1.17]
Wang et al. 2008	Postoperative Nausea and Vomiting	Absence of Nausea and Vomiting	21/26	18/26	1.17 [0.85, 1.60]
Turgut et al. 2007	Postoperative Nausea	Absence of Nausea	34/50	18/50	1.89 [1.25, 2.86]
Turgut et al. 2007	Postoperative Vomiting	Absence of Vomiting	37/50	21/50	1.76 [1.22, 2.54]
Heazell et al. 2006	Length of Hospitalization	Less than 4 Days in the Hospital	29/40	22/40	1.32 [0.94, 1.85]
Samad et al. 2003	Postoperative Nausea and Vomiting	Absence of Nausea and Vomiting	25/25	25/25	1.00 [0.93, 1.08]
Alkaissi et al. 2002	Postoperative Nausea and Vomiting	Absence of Nausea, Vomiting or Rescue Medicine	90/135	86/139	1.08 [0.90, 1.29]
Agarwal et al. 2000	Postoperative Nausea	Absence of Nausea	82/100	80/100	1.02 [0.90, 1.17]
Agarwal et al. 2000	Postoperative Vomiting	Absence of Vomiting	93/100	91/100	1.02 [0.94, 1.11]
Harmon et al. 2000	Postoperative Nausea and Vomiting	Absence of Nausea and Vomiting	30/47	16/47	1.88 [1.19, 2.95]
Harmon et al. 1999	Postoperative Nausea and Vomiting	Absence of Nausea and/or Vomiting	42/52	30/52	1.40 [1.07, 1.83]
Alkaissi et al. 1999	Postoperative Nausea and Vomiting	Absence of Nausea, Vomiting or Rescue Medicine	11/20	11/20	1.00 [0.57, 1.75]
Fan et al. 1997	Postoperative Nausea and Vomiting	Absence of Nausea and Vomiting	83/108	54/92	1.31 [1.07, 1.60]
Bayreuther et al. 1994	Morning Sickness	Symptom Reduction	10/15	5/15	2.00 [0.90, 4.45]
**Type 3** Pseudo-Intervention at the Same Acupoints as Intervention Group: Manual Light-Touch without Acupressure					
Kashanian & Shahali 2010	Cesarean Delivery	No Need for Cesarean Delivery	54/60	35/60	1.54 [1.23, 1.94]
Hjelmstedt et al. 2010	Use of Analgesics	No Need for Analgesics	36/71	37/71	0.97 [0.71, 1.34]
Lee et al. 2004	Use of Analgesics	No Need for Analgesics	31/36	29/39	1.16 [0.92, 1.45]
**Type 4** Pseudo-Intervention at the Same Acupoints as Intervention Group: Placebo Acupressure Devices					
Nilsson et al. 2015	Postoperative Nausea and Vomiting	Absence of Nausea and Vomiting	24/43	31/52	0.94 [0.66, 1.33]
Molassiotis et al. 2013	Chemotherapy-induced Nausea	No Nausea Experience	41/126	36/118	1.07 [0.74, 1.55]
Molassiotis et al. 2013	Chemotherapy-induced Vomiting	No Vomiting Experience	85/126	80/118	1.00 [0.84, 1.18]
Noroozinia et al. 2013	Postoperative Nausea and Vomiting	Absence of Nausea and Vomiting	76/76	72/76	1.06 [1.00, 1.12]
Soltanzadeh et al. 2012	Postoperative Nausea	Absence of Nausea	29/40	22/40	1.32 [0.94, 1.85]
Soltanzadeh et al. 2012	Postoperative Vomiting	Absence of Vomiting	32/40	25/40	1.28 [0.96, 1.70]
White et al. 2012	Postoperative Nausea	Absence of Nausea	30/50	26/50	1.15 [0.81, 1.64]
White et al. 2012	Postoperative Vomiting	Absence of Vomiting	45/50	37/50	1.22 [1.01, 1.47]
Ho et al. 2006	Intraoperative Nausea	Absence of Nausea	20/55	16/55	1.25 [0.73, 2.15]
Ho et al. 2006	Intraoperative Vomiting	Absence of Vomiting	43/55	40/55	1.07 [0.87, 1.33]
Klein et al. 2004	Postoperative Nausea	Absence of Nausea	50/75	50/77	1.03 [0.82, 1.29]
Klein et al. 2004	Postoperative Vomiting	Absence of Vomiting	63/75	62/77	1.04 [0.90, 1.21]
Schultz et al. 2003	Postoperative Nausea	Mild Nausea	14/25	14/21	0.84 [0.53, 1.33]
Dent et al. 2003	Post-MI Nausea and Vomiting	Absence of Nausea and Vomiting	55/95	48/98	1.18 [0.91, 1.54]
Duggal et al. 1998	Postoperative Nausea	Absence of Nausea	53/122	42/122	1.26 [0.92, 1.73]
Duggal et al. 1998	Postoperative Vomiting/Retching	Absence of Vomiting/Retching	72/122	66/122	1.09 [0.88, 1.36]
Lewis et al. 1991	Postoperative Vomiting	Absence of Vomiting	9/31	9/33	1.06 [0.49, 2.33]
**Type 6** Sham Acupressure at Non-Therapeutic Acupoints (Irrelevant): Acupressure Bands or Other Devices					
Nordio & Romanelli 2008	Melatonin Metabolite	Normal Melatonin Rhythm	13/18	5/15	2.17 [1.00, 4.68]

**TAG**: True acupressure group; **SAG**: Sham acupressure group; **MI**: Myocardial infarction

**#**: Outcome used for response rate estimation

#### Overall Assessment

Sixty-four studies reported treatment outcomes between the true and sham acupressure groups. For the main outcomes, 64.1% (41/64) reported that true acupressure was significantly superior to sham intervention, and 12.5% favored the true procedure but the difference did not reach statistical significance, while there were 13 studies (20.3%) which found no difference between groups. In addition, the sham acupressure was found to be more effective than true intervention in two trials (3.1%) [70, 74] with one even reaching statistical significance [70]. Among all the included studies, 12 compared the effects of acupressure (true/sham) with the study arms using standard methods of care. In four trials [23, 29, 32, 71], true acupressure was shown to be more effective than sham treatment and/or standard care, while no difference was found between sham acupressure and standard care. In another seven studies [26, 28, 45, 47, 51, 60, 64], both true and sham procedures were found to be superior to conventional care with or without statistical significance, while one trial [53] found no difference among groups. Thirty-four articles which provided dichotomous data were included for calculating responder rate ratio, and the majority (27 studies) supported the superiority of true acupressure compared with sham intervention, with the responder rate ratio (as measured by RR) greater than 1.0.

#### Subgroup Analysis

Subgroup analysis was performed based on different sham alternatives (**Table 4**). Fourteen out of 18 studies (77.8%) employing type 1 sham procedure favored true acupressure. For type 2 studies, 70% (14/20) supported the superiority of the true intervention. Of the 14 studies using pseudo-acupressure devices (type 4), nine (64.3%) reported that true acupressure was more effective than sham control, three (21.4%) found no difference between groups, and two (14.3%) favored sham acupressure. Number of studies for sham type 3, 5 and 6 were limited, but all supported the superiority of true intervention. Responder rate ratios were also separately analyzed for different sham acupressure types and the true intervention groups were found to be more effective than the sham comparisons in the majority of the analyzed trials within each sham acupressure control (**Table 6**).

Subgroup analyses were also applied for different types of health conditions and treatment duration (**Table 5**). Descriptive analysis showed that true acupressure can be an effective approach in managing postoperative and pregnancy-related nausea and/or vomiting, perioperative/prehospital pain, labor pain, sleep disturbances, respiratory disorders, anxiety, postoperative gastrointestinal dysfunctions and motion sickness. Studies on primary dysmenorrhea and chemotherapy-induced nausea and vomiting reported contradictory findings, where two studies [57, 77] supported the superiority of true acupressure, while another two [17, 64] stated no difference between groups. Results from a single study indicated that the true intervention was better than sham control in treating urodynamic stress incontinence [23], traumatic brain injury [24], cancer-related fatigue [16] and myocardial infarction-related nausea and vomiting [71], and in maintaining women’s general health [25] and stroke survivors’ cardiovascular functions [27]. The only study on stress reduction failed to prove the superiority of true acupressure [20]. For each subgroup under “treatment duration”, more than half (68.75% to 83.3%) supported that true acupressure was more effective than sham intervention (**Table 5**). Further investigation on acupressure frequency and intensity was not feasible due to insufficient information and absence of available data.

### Dropout Rates and Adverse Events associated with Acupressure

In 26 studies, all the randomized subjects completed the study. Thirty-four studies reported participants’ dropout, however, 11 of them only provided the total dropout number but failed to specify the number in each study arm [19, 32, 47, 51, 53, 62, 70, 72, 75, 79, 80]. Reasons for dropout were diverse and included changing treatment protocol, refusing to continue with the study, suffering from adverse events, or death, etc. The total dropout rate was 6.2%, with the true intervention groups 6.1% and the sham groups 6.4%. Dropout rates also varied significantly among different sham alternatives. The lowest was found in type 3 trials, with the total dropout rate being 1.22% (true acupressure 0.8% and sham comparison 1.6%). Chi-square tests showed that there was no difference of the dropout rates between groups (**Table 7**).

**Table 7:** Summary of Dropout Rate Reported in the Included Studies.

		Number of Dropout/Sample Size (Dropout Rate, %)			
Types of Sham Control	No. of Studies#	TAG	SAG	Total	Chi-Squared Test
**Type 1**	15	51/457 (11.2%)	37/450 (8.2%)	88/907 (9.7%)	χ² = 2.233, df = 1, P = 0.135
**Type 2**	15	20/895 (2.2%)	24/882 (2.7%)	44/1777 (2.5%)	χ² = 0.435, df = 1, P = 0.509
**Type 3**	6	2/246 (0.8%)	4/246 (1.6%)	6/492 (1.2%)	χ² = 0.169, df = 1, P = 0.681
**Type 4**	10	64/729 (8.8%)	74/716 (10.3%)	138/1445 (9.55%)	χ² = 1.013, df = 1, P = 0.314
**Type 5**	2	6/46 (13.0%)	7/46 (15.2%)	13/92 (14.1%)	χ² = 0.090, df = 1, P = 0.765
**Type 6**	1	2/20 (10.0%)	5/20 (25.0%)	7/40 (17.5%)	χ² = 0.693, df = 1, P = 0.405
**All Types**	49	145/2393 (6.1%)	151/2360 (6.4%)	296/4753 (6.2%)	χ² = 0.234, df = 1, P = 0.629

**Type 1**: Sham acupressure at non-acupoints by manual pressure; **Type 2**: Sham acupressure at non-acupoints by employing acupressure devices; **Type 3**: Pseudo-intervention at the same acupoints as true treatment arm by manual light-touch; **Type 4**: Pseudo-intervention at the same acupoints as true treatment arm by using placebo devices; **Type 5**: Manual acupressure at non-therapeutic (irrelevant) acupoints; **Type 6**: Sham acupressure at non-therapeutic (irrelevant) acupoints by adopting acupressure devices

**#** Number of studies which were available for dropout rate calculation

There were 24 studies that observed potential adverse events associated with acupressure, among which, 13 reported no adverse events, and the other 11 reported acupressure device-related harm data including local swelling and redness, discomfort and tenderness at the wrist, and paresthesia, etc. No serious adverse events were noted. There was one study [67] that detected adverse events such as constipation, headache, fatigue and drowsiness, but information was insufficient to judge whether these were caused by acupressure. No study assessed the causality between the acupressure and the reported adverse events.

## Discussion

This study analyzed 66 trials with six sham acupressure types. The findings indicated that “non-acupoint” was the most frequently selected sham acupoint and an acupressure device was the commonly adopted approach for sham intervention. Meanwhile, our findings supported that acupressure was a beneficial method in managing a variety of health problems, and the effect was found to be more effective in true acupressure than that in sham procedure. Because of the significant clinical heterogeneity among the included studies, the relationship between sham acupressure methods and the treatment outcomes is not fully conclusive at this stage.

Our findings showed that true acupressure was more effective than sham acupressure, and the effect of sham intervention was somewhat better than that in the standard care arms. These results support the specific benefits of true acupoint stimulation, but also indicate that sham acupressure more or less produces some specific or non-specific treatment effects. Sham acupressure may be associated with larger effects than control groups adopting standard methods of care. Placebo effects of sham acupoint stimulation may be related to patients’ expectation of treatment, and ventral striatum and prefrontal cortex are commonly believed to be involved in the activation of the reward system which contributes to a feeling of symptom improvement during intervention [82]. Previous studies indicated that “physical placebos”, including sham acupoint stimulation (e.g. acupuncture), could result in larger effects over non-treatment arms than drug placebos [83, 84]. Reasons for the relatively larger effects in sham acupoint stimulation are complicated, and from a psychological perspective, any forms of acupoint stimulation could be viewed as an emotional focused therapy which more or less produces some treatment effects [82]. However, whether the effects produced by sham acupoint stimulation are only non-specific placebo effects or are mixed with any specific therapeutic benefits, we still cannot reach a definite conclusion at present.

Similar to the results from the descriptive analysis, the exploratory analysis of the responder rate ratios also showed that true acupressure was more effective than sham comparisons regardless of the sham acupressure types. However, these findings should be interpreted prudently because the responder rate ratio only provides a rough summary of the study results, and the dichotomous data we used were not the main measures in some of the analyzed trials. Meanwhile, even within the subgroup of each sham acupressure method, clinical heterogeneities (e.g. differences in the health conditions, patient characteristics, intervention duration and outcome measures, etc.) were still considerable in the analyzed trials. In addition, the number of studies included within each subgroup was quite imbalanced among the sham types, with type 2 and 4 respectively gathering more than 10 studies, whereas other sham modalities only including no more than three studies.

In the analyzed studies, non-acupoint was the most frequently adopted sham acupoint approach used. Non-acupoint is a popular sham modality because generally it would not create any therapeutic effects. However, we are not sure whether all the so-called “non-acupoints” in our analyzed trials are real non-acupoints because only few of them pointed out that the selected non-acupoints were situated away from the meridians, while in the remaining studies, the relation between the non-acupoints and their nearest active acupoints and/or meridians was not provided. Meanwhile, researchers should prudently select the non-acupoints because currently there are more than 2000 established extra-points which are not linked with meridians, and this cannot exclude the possibility that the so called “non-acupoints” in some trials may be unintentionally therapeutic points in nature [85].

In addition to the non-acupoints, true acupoints were also commonly used as sham acupressure. The acupoints were the same as the active intervention groups but were only provided with light tough or totally received no stimulation. It is believed that light stimulation on the true acupoints would not activate the *deqi* sensation, and thus, avoid the generation of specific treatment effects [6, 85]. But recent studies on pain concluded that light stimulation on the skin could evoke the activity of the cutaneous afferent nerves and/or the insular region of the brain, and may result in some augmented therapeutic benefits [4, 86]. Also, patients with pain conditions could experience similar treatment effects when the selected true and sham acupoints located at the same or nearby myotome [4, 87]. Light touch may not be a proper sham approach for studies on pain management. Also, light stimulation was deemed inadequate to maintain blinding of the participants, especially for those with previous acupressure experience. Light touch does not exert additional pressure and avoids evoking the *deqi* sensation. When the intervention is observed by participants, it may be easy for them to judge that they are not receiving the “true” acupressure.

Non-therapeutic acupoints were used as the sham intervention in five of the included studies. Regarding to the location of the non-therapeutic acupoint, it is recommended to be situated near the selected active acupoint to achieve proper blinding, but is also required to avoid being on the same meridian as in the true intervention. In one trial which focused on pre-hospital analgesia for patients with distal radius fracture, *jianliao* (TE14) was employed as one of the sham acupoints, and acupressure intensity was equal in both groups [78]. However, this kind of sham design might not be adequate enough because acupressure at any of the body acupoints could contribute to some pain relieving effects. In the meantime, *jianliao* is mainly used for treating shoulder and back pain, although not specific for distal radius fracture, stimulation of *jianliao* might somewhat produce some positive impact on the pain management of the whole body. According to the “holism concept” of acupuncture theory, every single stimulation of the acupoint could result in comprehensive responses of the human body and subsequently generate specific and/or non-specific effects [85]. That is to say, pressing irrelevant acupoints may also create some treatment effects for the targeted condition, especially when the sham intervention receives the same acupressure intensity as in the active intervention arm.

Acupressure devices can be an adequate method for retaining blinding. In our analyzed studies, acupressure bands with or without the pressure button were commonly incorporated as sham interventions. The same or identical equipment used in both groups makes the blinding of participants and outcome assessors possible, and blinding of care providers can also be achieved if they are not involved in the application of the acupressure devices. In type 2 sham designs, most of the trials employed acupressure equipment on non-acupoints and used the same acupressure intensity as in the true intervention arms. However, as mentioned above, acupressure at non-acupoints might induce some unexpected treatment effects, and thus makes the type 2 design more effective than its own placebo effects. Type 4 designs with pseudo-devices could be viewed as a good sham mode because those do not apply additional pressure to the selected acupoints which could minimize the potential therapeutic effects at a greater extend. Also, the appearance looks indistinguishable from the true acupressure tools which could keep the blinding at a satisfactory level, particularly for acupressure-naive participants. Unfortunately, tools such as acupressure bands currently are only applicable for acupoints located on the arms or legs, and when a study adopts too many acupoints, using acupressure equipment nearly becomes impossible.

Participants’ dropout rates were similar between groups. Although reasons for dropout varied among trials, no participants were reported dropping out because of being allocated to sham group. Meanwhile, this review found that the type 3 control had the lowest dropout rate. However, the low rate seemed not to be caused by the sham intervention itself but by the nature of the included health problems. Among the six studies with the type 3 sham approach, more than half were studying pregnant women preparing for delivery and the acupressure only lasted several minutes.

For studies that observed potential adverse events associated with acupressure, no such events were found in more than half of the studies. Adverse events related to acupressure identified in the remaining trials were generally mild and transient. Reported adverse events were associated with acupressure devices, which were mostly attributed to wearing of the equipment for a longer time. Loosening the bands temporarily during the intervention could help to reduce the side effects to some extent. Acupressure seems to be a relatively safe approach for acupoints-stimulation. However, it is noted that none of the included trials assessed the causality between the acupressure and the reported adverse events, and standard causality assessment tools (e.g. The WHO-Uppsala Monitoring Centre System for Standardized Case Causality Assessment) were failed to be applied in the analyzed studies, which make it difficult to judge whether these adverse events are really caused by the acupressure intervention or not.

Some limitations were identified in the analyzed studies which could affect the reliability of our results. Firstly, true and sham acupressure protocols were described unclearly in some studies. Relationship and distance between the non-acupoint and the nearest meridian were seldom reported. Information on intervention frequency and intensity were also provided insufficiently in some trials, which made the subgroup analyses on the acupressure frequency and intensity impossible. Secondly, subjective outcomes were utilized in more than half of the articles, which produced a possibility for exaggerating the treatment effects of the intervention, especially for studies with inadequate blinding and/or allocation concealment [88]. Thirdly, methodological flaws still existed among studies. Assessment of the credibility of blinding is essential in clinical trials for evaluating the successfulness of blinding design, especially for trials with a placebo/sham control arm, as inadequate blinding could contribute to performance and/or detection biases, which could exaggerate the potential specific/non-specific treatment effects of the placebo/sham intervention. However, credibility of blinding was not tested in all the trials, and for some studies to which the blinding of care providers could have been achieved (e.g. studies using identical acupressure bands), such a design also failed to be incorporated. Moreover, Chinese syndrome differentiation was not employed to guide subject recruitment, acupoint identification, and acupressure-technique selection in studies using Chinese acupressure, which may downgrade the feasibility and reliability of the intervention protocols to some degree.

The study objective which focused on whether different types of sham procedure yield different therapeutic outcomes was difficult to achieve in this review as limited homogeneous data could be accessed for conducting subgroup analysis on the same health condition within each sham acupressure modality. Descriptive analysis only provided a preliminary summary of the therapeutic effects of acupressure. The exploratory analysis of the responder rate ratio was unable to always be consistent with the finding drawn from the study main outcomes with continuous variables as responder rate ratio was only a rough estimation and dichotomous data were not the main outcomes for some studies. Meanwhile, even within single sham alternative, trials still showed heterogeneity in a number of aspects, such as the differences in patient characteristics, treatment duration and the outcome measures, etc., all of which made the interpretation of results a difficult task. In addition, our findings at the current stage could not evaluate whether there were any sham designs that had produced additional treatment effects over and above their own placebo effects. Finally, although we have made efforts to locate as much eligible studies as possible, the final included studies were only in English and Chinese. Several articles written in languages other than English or Chinese were excluded, inevitably leading to some language bias.

## Implications for Future Research and Practice

Verbal and non-verbal interactions between practitioners/researchers and participants should be pre-standardized to avoid increasing participants’ expectations to the treatment which may exaggerate the non-specific psychological effects of the treatment [89, 90]. In studies utilizing an acupressure device, it is suggested that researchers and care providers are not involved in the application of acupressure equipment in order to retain satisfactory blinding. Furthermore, it would be better for future studies to recruit participants without any previous experience in acupressure. Acupressure-naive subjects do not have any experience of *deqi* sensation, which may increase the chance of reaching successful blinding of participants [6]. To equalize the treatment expectations among participants, if possible, participants’ characteristics such as educational background, religion, and lifestyle, etc. should be kept similar at the baseline, as these elements are considered to be potential social-psychological factors that could affect participants’ expectations to treatment [85].

Selection of the sham methods should be in accordance with the study objectives and the actual requirements of the intervention and participants, as well as the nature of the disease being treated. For example, if a study intends to test the specificity of some particular acupoints, the true and sham acupressure protocols should be the same with the only difference in the selected acupoints. To minimize potential therapeutic effects of sham interventions and to maintain a satisfactory blinding design, non-acupoints located near the targeted true acupoints are generally recommended but the definite locations should be identified with caution, and an acupoint detector (a commonly utilized device for accurately locating effective body/auricular acupoints) could be considered to distinguish ineffective non-acupoints from active true acupoints. For acupressure intensity, future studies are suggested to exert only light or no stimulation to the sham acupoints to prevent the generation of the *deqi* sensation. Particularly, acupoint stimulation should be avoided in pain studies adopting sham acupressure. While for studies using single sham acupoints on hands or legs, it is suggested to apply identical acupressure devices on the same acupoints as in the true intervention groups without any stimulation.

To facilitate the blinding of participants and/or care providers, some methods could be considered to prevent them from observing the treatment procedure, for instance, letting participants wear eye patches when administering the intervention [91], or using bandages to cover the wrist bands during surgical acupressure trials. Moreover, some studies also pointed out that the effects of acupoint stimulation can be reflected on the neural activities in the limbic system and subcortical structures which provides a possible direction for future research to use the functional magnetic resonance imaging (fMRI) to adequately evaluate sham procedures based on the neural activities of the brain [92,93].

As subjective outcomes (e.g. questionnaire and visual analogue scale, etc.) could be affected by the participants’ expectations to treatment, which subsequently exaggerates therapeutic effects, future studies could consider including some objective measures. Some scholars point out that instead of using the word of “sham” or “placebo”, informing participants that the aim of study is to compare two different treatment procedures would be more credible to reach a successful blinding of study subjects [2, 94]. This kind of incomplete informed consent may be in debate for ethical reasons [2]. However, according to the results of a nested qualitative study within a RCT using acupressure (both true and sham) for controlling chemotherapy-induced nausea, it was acceptable for the study participants to receive this kind of consent process [94]. Meanwhile, from the findings of this review as well as other studies on sham acupoint stimulation [92, 95], sham interventions can also contribute to symptom improvement, and thus, it seems plausible to view the sham acupressure as a non-specific treatment approach in nature. Finally, the methodological quality of future trial needs to be improved. More detailed information on the theoretical background of the selection and identification of true and sham acupoints should be provided. Treatment duration, number of sessions, and acupressure intensity should be described in detail in the acupressure protocols. Even if it is difficult to incorporate blinding of the practitioner in manual acupressure trials, a double-blind design for both participants and outcome assessors is still possible. Credibility of blinding should also be tested to investigate the successfulness of blinding design and subsequent to identify the potential risk of performance and/or detection biases. Related guidelines such as the CONSORT [96] and STRICTA [97] guidelines should be followed in the development and reporting of acupressure trials.

## Conclusions

A great diversity of sham acupressure controls have been used in the literature. A solid conclusion whether different sham alternatives are related to different treatment outcomes cannot be derived because of the significant clinical heterogeneity among the analyzed trials. Based on the findings of this systematic review, non-acupoints are generally recommended but the definite locations should be identified with caution. For studies using single sham acupoints on hands or legs, it is suggested to apply identical acupressure devices on the same acupoint as in the active intervention without any stimulation. While for studies on pain, stimulation of sham acupoints should be avoided.

## Supporting Information

S1 File PRISMA Checklist. (PDF)

S2 File Systematic Review Protocol. (PDF)

S1 Table Selected Searching Strategies. (PDF)

S2 Table Excluded Acupressure Trials due to High Risk of Bias. (PDF)

S3 Table Risk of Bias Assessment of the Included Trials. (PDF)
