# Supplementary material for: Sham Acupressure Controls Used in Randomized Controlled Trials: A Systematic Review and Critique
Source: PLoS One. 2015 Jul 15;10(7):e0132989. doi: 10.1371/journal.pone.0132989 (PMC4503717; doi:10.1371/journal.pone.0132989)
Supplement: S2 File — (PDF) [file pone.0132989.s002.pdf]

Study Protocol: Sham Controls Used in Randomized Controlled Trials of Acupressure: A Critical Literature Analysis

Jing-Yu Tan RN, PhD(c)
Lorna K.P. Suen RN, PhD
Tao Wang RN, MScN
Alexander Molassiotis RN, PhD [Corresponding Author]

Aims

- To summarize the commonly utilized sham acupressure procedures in existing randomized controlled trials.
- To investigate whether there are any differences in the treatment outcomes between the true acupressure groups and the sham acupressure groups among all the included trials.
- To assess whether different types of sham acupressure controls are associated with different therapeutic outcomes.
- To identify directions for the future development of an adequate sham acupressure mode.

Methods

Data Sources

- **Electronic Search in 13 Databases**
  - PubMed; EMBase; Cochrane Central Register of Controlled Trials (CENTRAL); CINAHL; Allied and Complementary Medicine (AMED); PsycINFO; Thomson Reuters Web of Science; Science Direct; Foreign Medical Journal Service (FMJS); China National Knowledge Infrastructure (CNKI); WanFang Data; Chinese Scientific Journal Database (CQVIP); Chinese Biomedical Literature Database (CBM)
- **Manual Search on Eight Chinese CAM Journals (Issues Published within the latest 3 Years)**
  - Chinese Acupuncture & Moxibustion; Journal of Traditional Chinese Medicine; China Journal of Traditional Chinese Medicine and Pharmacy; Journal of Beijing University of Traditional Chinese Medicine; Journal of Nanjing University of Traditional Chinese Medicine; Chinese Journal of Integrated Traditional and Western Medicine; Chinese Journal of Information on Traditional Chinese Medicine; Chinese Journal of Basic Medicine in Traditional Chinese Medicine
- **Reference Lists of Systematic Reviews on Acupressure**
- **Reference Lists of Included Trials**

Search Terms

- **Mesh Terms**
  - [English] acupressure or (“acupuncture point” and “wrist”)
  - [Chinese] 穴位按压
- **Entry Terms, Key Words or Free Words**
  - [English] acupress* or acustim* or (“acupunct*” and “pressure*”)
  - [English] shiatsu or shiatzu or “zhi ya” or “chih ya”
  - [English] wristband* or (“wrist” and “band*”) or (“relief” and “band*”) or (“sea” and “band*”)
  - [Chinese] 穴位按压 or 穴位按摩 or 穴位贴压 or 提针疗法 or 指压 or 指针

Type of Studies

- Randomized controlled trials (RCTs) comparing true acupressure with sham acupressure.
- For studies with more than two active treatment arms, only true and sham acupressure groups will be included for analysis.
- For studies including another control group with standard care, data from the standard care arm will also be extracted for analysis.
- Study protocols and RCTs on body acupuncture including manual acupuncture, electro-acupuncture, and laser-acupuncture, as well as all types of auricular therapy will be excluded.

Methodological Quality Assessment of Included Trials

- “Risk of Bias Assessment Criteria” developed by the Cochrane Back Review Groups [1]:
  - “Was the method of randomization adequate?”
  - “Was the treatment allocation concealed?”
  - “Was the patient blinded to the intervention?”

- “Was the care provider blinded to the intervention?”
  - “Was the outcome assessor blinded to the intervention?”
  - “Was the drop-out rate described and acceptable?”
  - “Were all randomized participants analyzed in the group to which they were allocated?”
  - “Are reports of the study free of suggestion of selective outcome reporting?”
  - “Were the groups similar at baseline regarding the most important prognostic indicators?”
  - “Were co-interventions avoided or similar?”
  - “Was the compliance acceptable in all groups?”
  - “Was the timing of the outcome assessment similar in all groups?”
- Each item will be evaluated as “yes (low risk of bias)”, “unclear (unclear risk of bias)” and “no (high risk of bias)”. The overall methodological quality of one single trial will be evaluated as “low risk of bias” when at least six items are rated as “yes” and no serious flaws are identified (e.g., 80% dropout rate was identified in one study group) [1]. Only studies rated as “low risk of bias” can be included for analysis.

Data Extraction

- First author, year of publication, country of origin, full name of journal, and study design and setting
- Participant characteristics (age, gender, sample size, dropout rate, diagnostic criteria, inclusion and exclusion criteria, and reason for acupressure)
- True and sham acupressure protocols (practitioner, equipment, selected acupoints, frequency and duration of treatment, number of treatment sessions, intensity and frequency of acupressure, etc.)
- Primary outcome(s) and results of therapeutic effects
- Acupressure-related adverse events
- Methodological quality (items in “Risk of Bias Assessment Criteria” plus “credibility of blinding in study subjects”)

Therapeutic Effects of Acupressure (Qualitative and Quantitative Analysis)

- Overall assessment <u>(Descriptive Analysis Only)</u> **(Table 2)**
  - Whether acupressure is effective comparing with wait-list control/usual care control/non-treatment control?
  - Whether true acupressure is superior to sham acupressure?
- Subgroup analysis will be conducted based on the following criteria:
  - Different types of sham acupressure controls **(Table 3)**
  - Different types of health problems (if possible)
  - Different treatment durations (if possible)
- Responder rate ratios (responder rate in true acupressure group/responder rate in sham acupressure group) with 95% confidence interval (CI) will be calculated for trials presenting dichotomous variables (random effects model, RevMan 5.3). Definition of response will be based on each individual study.
- Chi-square analysis will be performed to investigate the difference in dropout rates between the true and sham acupressure groups (IBM SPSS Statistics 20).

Table 2 Vote Counts of Results of Therapeutic Effects [2]

Classification	Description
+	True Acupressure is Significantly Better than Sham Control for the Primary Outcome(s)
(+)	Trend of the Primary Outcome(s) in Favor of True Acupressure but without Statistical Significance
0	No Difference between True and Sham Acupressure
(-)	Trend of the Primary Outcome(s) in Favor of Sham Acupressure but without Statistical Significance
-	Sham Intervention is Significantly Better than True Acupressure for the Primary Outcome(s)

Table 3 Classification of Subgroups for Sham Acupressure Controls [2-4]

**Type 1**	Sham Acupressure at Non-acupoints by Manual Pressure
**Type 2**	Sham Acupressure at Non-acupoints by Employing Acupressure Devices
**Type 3**	Pseudo-intervention at the Same Acupoints as True Treatment Arm by Manual Light-Touch
**Type 4**	Pseudo-intervention at the Same Acupoints as True Treatment Arm by using Placebo Devices
**Type 5**	Manual Acupressure at Non-Therapeutic (Irrelevant) Acupoints
**Type 6**	Sham Acupressure at Non-Therapeutic (Irrelevant) Acupoints by Adopting Acupressure Devices
**Others**	Combination of Above Two or More Approaches

Orientations for Analysis and Discussion

Overall Assessment

- Is acupressure (both true and sham intervention) effective comparing with wait-list control/usual care control/non-treatment control?
- Is true acupressure superior to sham acupressure?
- Does acupressure also create some non-specific physiological effects or non-specific psychological (placebo) effects?
- The clinical value of acupressure even if this kind of approach may only create non-specific effect.

Subgroup Assessment

- Whether different types of sham acupressure approaches are associated with different therapeutic outcomes.
- Which type of sham acupressure is the most appropriate sham control design (if any)?
- Strengths and weaknesses of each type of sham acupressure design included in this review.

Hints for Develop an Appropriate Sham Control in Acupressure Trials (From both Theoretical and Practical Aspects**)**

- Selected acupoints,
- Intervention procedure (manipulation/pressure technique, frequency, intensity, and duration of treatment),
- Professional skills of acupuncturists,
- Interactions between acupuncturist and patient,
- Patients' expectation,
- Outcome measures (subjective or objective),
- Methodological issues (sample size calculation, blinding of study participants and/or data assessors, and allocation concealment),
- Other issues.

References

[1] Furlan AD, Pennick V, Bombardier C, van Tulder M. (2009). 2009 updated method guidelines for systematic reviews in the Cochrane Back Review Group. Spine, 34(18): 1929-1941.

[2] Dincer F, Linde K. (2003). Sham interventions in randomized clinical trials of acupuncture: a review. Complementary Therapies in Medicine, 11(4): 235-242.

[3] Zhang CS, Yang AW, Zhang AL, May BH, Xue CC. (2014). Sham control methods used in ear-acupuncture/ear-acupressure randomized controlled trials: a systematic review. The Journal of Alternative and Complementary Medicine, 20(3): 147-161.

[4] Zhang H, Bian Z, Lin Z. Are acupoints specific for diseases? A systematic review of the randomized controlled trials with sham acupuncture controls. Chinese Medicine, 2010, 5(1).
