# Supplementary material for: Sham Acupressure Controls Used in Randomized Controlled Trials: A Systematic Review and Critique
Source: PLoS One. 2015 Jul 15;10(7):e0132989. doi: 10.1371/journal.pone.0132989 (PMC4503717; doi:10.1371/journal.pone.0132989)
Supplement: S1 Table — (PDF) [file pone.0132989.s003.pdf]

Supporting Information Table S1

Table S1 Selected Searching Strategies

Items	Search Strategy	Results
	**PubMed**	
**#1**	“acupressure”[MeSH Terms]	453
**#2**	("acupuncture points"[MeSH Terms]) AND "wrist"[MeSH Terms]	23
**#3**	((((((((((acupress*[Title/Abstract]) OR (acustim*[Title/Abstract]) OR (acupunct*[Title/Abstract] AND pressure*[Title/Abstract])) OR shiatsu[Title/Abstract]) OR shiatzu[Title/Abstract]) OR “zhi ya” [Title/Abstract]) OR “chih ya” [Title/Abstract]) OR wristband*[Title/Abstract]) OR (wrist [Title/Abstract] AND band*[Title/Abstract])) OR (relief[Title/Abstract] AND band*[Title/Abstract])) OR (sea[Title/Abstract] AND band*[Title/Abstract]) OR “acupoint massage” [Title/Abstract])	3122
**#4**	#1 OR #2 OR #3	3258
**#5**	(((((((("randomized controlled trial"[Publication Type]) OR "controlled clinical trial"[Publication Type]) OR "ramdomized"[Title/Abstract]) OR "ramdomised"[Title/Abstract]) OR "placebo"[Title/Abstract]) OR "sham"[Title/Abstract]) OR "randomly"[Title/Abstract]) OR "trial"[Title/Abstract]) OR "groups"[Title/Abstract]	2014382
**#6**	(animals[MeSH Terms] NOT (humans[MeSH Terms] AND animals[MeSH Terms]))	3902103
**#7**	#5 NOT #6	1657325
**#8**	#4 AND #7	843
	**EMBase**	
**#1**	'acupressure'/exp	1447
**#2**	acupress*:ab,ti OR acustim*:ab,ti OR (acupunct* NEAR/10 pressure*):ab,ti OR shiatsu:ab,ti OR shiatzu:ab,ti OR (zhi NEAR/3 ya):ab,ti OR (chih NEAR/3 ya):ab,ti OR wristband*:ab,ti OR (wrist NEAR/3 band*):ab,ti OR (relief NEAR/3 band*):ab,ti OR (sea NEAR/3 band*):ab,ti OR (acupoint* NEAR/3 massage):ab,ti	1497
**#3**	#1 OR #2	2105
**#4**	'controlled clinical trial'/exp OR 'single blind procedure'/exp OR 'double-blind procedure'/exp OR 'crossover procedure'/exp	503586
**#5**	random*:ab,ti OR crossover*:ab,ti OR (cross NEAR/3 over*):ab,ti OR placebo:ab,ti OR (doubl* NEAR/3 blind*):ab,ti OR (doubl* NEAR/3 mask*):ab,ti OR (singl* NEAR/3 blind*):ab,ti OR (singl* NEAR/3 mask*):ab,ti OR (trebl* NEAR/3 blind*):ab,ti OR (trebl* NEAR/3 mask*):ab,ti OR (tripl* NEAR/3 blind*):ab,ti OR (tripl* NEAR/3 mask*):ab,ti OR assign*:ab,ti OR allocat*:ab,ti OR volunteer*:ab,ti	1341777
**#6**	#4 OR #5	1508321
**#7**	'animal'/exp OR 'nonhuman'/exp OR 'animal experiment'/exp	20487702
**#8**	'human'/exp	15606399
**#9**	#7 AND #8	15304501
**#10**	#7 NOT #9	5183201
**#11**	#6 NOT #10	1338562
**#12**	#3 AND #11	739
	**Cochrane Central Register of Controlled Trials (CENTRAL)**	
**#1**	MeSH descriptor: [Acupressure] explode all trees	226
**#2**	MeSH descriptor: [Acupuncture Points] explode all trees	1051
**#3**	MeSH descriptor: [Wrist] explode all trees	236
**#4**	#2 AND #3	12
**#5**	#1 OR #4	230
**#6**	acupress* or acustim* or (acupunct* near/10 pressure*) or shiatsu or shiatzu or (zhi near/3 ya) or (chih near/3 ya) or wristband* or (wrist near/3 band*) or (relief near/3 band*) or (sea near/3 band*) or (acupoint* near/3 massage):ti,ab,kw (Word variations have been searched)	688
**#7**	#5 OR #6	689
**#8**	In Trials	635
	**Chinese Biomedical Literature Database (CBM)**	
**#1**	主题词:穴位按压△/不扩展/全部副主题词 -限定: 临床试验，随机对照试验，多中心研究，人类	1099
**#2**	中文标题:穴位按压 or 穴位按摩 or 穴位贴压 or 指压疗法 -限定:临床试验，随机对照试验，多中心研究，人类	436
**#3**	#1 OR #2	1170
