# Supplementary material for: Sham Acupressure Controls Used in Randomized Controlled Trials: A Systematic Review and Critique
Source: PLoS One. 2015 Jul 15;10(7):e0132989. doi: 10.1371/journal.pone.0132989 (PMC4503717; doi:10.1371/journal.pone.0132989)
Supplement: S2 Table — (PDF) [file pone.0132989.s004.pdf]

Supporting Information Table S2

Table S2 Excluded Acupressure Trials due to High Risk of Bias

No.	Trials
1	Agarwal A, Ranjan R, Dhiraaj S, Lakra A, Kumar M, Singh U. Acupressure for prevention of pre-operative anxiety: a prospective, randomised, placebo controlled study. Anaesthesia, 2005, 60(10), 978-981.
2	Barsoum G, Perry EP, Fraser IA. Postoperative nausea is relieved by acupressure. J R Soc Med, 1990, 83(2), 86-89.
3	Bazarganipour F, Lamyian M, Heshmat R, Abadi MAJ, Taghavi A. A randomized clinical trial of the efficacy of applying a simple acupressure protocol to the Taichong point in relieving dysmenorrhea. Int J Gynaecol Obstet, 2010, 111(2), 105-109.
4	Borimnejad L, Arbabi N, Seydfatemi N, Inanloo M, Haghanii H. (2012). The Effects of Acupressure on Preoperative Anxiety Reduction in School Aged Children. Health Med, 2012, 6(7), 2359-2361.
5	Can Gürkan Ö, Arslan H. Effect of acupressure on nausea and vomiting during pregnancy. Complement Ther Clin Pract, 2008, 14(1), 46-52.
6	Chate, RAC. PC. 6 Acupressure for dental nausea: a preliminary report of a prospective randomised double blind clinical trial, part 1. Acupunct Med, 1997, 15(1), 6-9.
7	Chen ML, Lin LC, Wu SC, Lin JG. The effectiveness of acupressure in improving the quality of sleep of institutionalized residents. J Gerontol A Biol Sci Med Sci, 1999, 54(8), M389-M394.
8	De Aloysio D, Penacchioni, P. Morning sickness control in early pregnancy by Neiguan point acupressure. Obstet Gynecol, 1992, 80(5), 852-854.
9	Dibble SL, Luce J, Cooper BA, Israel J, Cohen M, Nussey B, Rugo H. Acupressure for chemotherapy-induced nausea and vomiting: a randomized clinical trial. Oncol Nurs Forum, 2007, 34(4), 1-8.
10	Dundee JW, Sourial FBR, Ghaly RG, Bell PF. P6 acupressure reduces morning sickness. J R Soc Med, 1988, 81(8), 456-457.
11	Felhendler D, Lisander B. Effects of non-invasive stimulation of acupoints on the cardiovascular system. Complement Ther Med, 1999, 7(4), 231-234.
12	Genç A, Can G, Aydiner A. The efficiency of the acupressure in prevention of the chemotherapy-induced nausea and vomiting. Support Care Cancer, 2013, 21(1), 253-261.
13	Hirš I, Lukić A, Fumić NN, Kekić M, Kotaran J. Acupressure and metoclopramide comparison in postoperative nausea and vomiting prevention on laparatomy patients. Acupuncture and Related Therapies, 2013, 1(4), 42-45.
14	Iqbal U, Khan A, Sheikh F. Whether Does Acupressure (p6) Prevent Nausea and Vomiting in Patients Undergoing Laparoscopic Surgery. Pakistan Journal of Medical and Health Sciences, 6(4):973-975.
15	Jones E, Isom S, Kemper KJ, McLean TW. Acupressure for chemotherapy-associated nausea and vomiting in children. J Soc Integr Oncol, 2007, 6(4), 141-145.
16	Kashefi F, Ziyadlou S, Khajehei M, Ashraf AR, Reza Fadaee A, Jafari P. Effect of acupressure at the Sanyinjiao point on primary dysmenorrhea: a randomized controlled trial. Complement Ther Clin Pract, 2010, 16(4), 198-202.
17	Maa SH, Gauthier D, Turner, M. Acupressure as an adjunct to a pulmonary rehabilitation program. J Cardiopulm Rehabil Prev, 1997, 17(4), 268-276.
18	Perkins P, Vowler SL. Does acupressure help reduce nausea and vomiting in palliative care patients? Pilot study. Palliat Med, 2008, 22(2), 193-194.
19	Pouresmail Z, Ibrahimzadeh R. Effects of acupressure and ibuprofen on the severity of primary dysmenorrhea. J Tradit Chin Med, 2002, 22(3), 205-210.
20	Sabouhi F, Kalani L, Valiani M, Mortazavi M, Bemanian M. Effect of acupressure on fatigue in patients on hemodialysis. Iran J Nurs Midwifery Res, 2013, 18(6), 429-434.
21	Tsay SL. Acupressure and fatigue in patients with end-stage renal disease–a randomized controlled trial. Int J Nurs Stud, 2004, 41(1), 99-106.
22	Windle PE, Borromeo A, Robles H, Ilacio-Uy V. The effects of acupressure on the incidence of postoperative nausea and vomiting in postsurgical patients. J Perianesth Nurs, 2001, 16(3), 158-162.
