# Supplementary material for: Sham Acupressure Controls Used in Randomized Controlled Trials: A Systematic Review and Critique
Source: PLoS One. 2015 Jul 15;10(7):e0132989. doi: 10.1371/journal.pone.0132989 (PMC4503717; doi:10.1371/journal.pone.0132989)
Supplement: S3 Table — (PDF) [file pone.0132989.s005.pdf]

Supporting Information Table S3

Table S3 Methodological Quality Assessment of the Included Studies

Study (Sort by Year of Publication)	Item 1	Item 2	Item 3	Item 4	Item 5	Item 6	Item 7	Item 8	Item 9	Item 10	Item 11	Item 12
1 Nilsson, et al. 2015	Yes	Yes	Yes	No	Yes	Yes	No	Yes	Yes	Yes	Unsure	Yes
2 Tang, et al. 2014	Yes	Unsure	Yes	No	Yes	Yes	Yes	Yes	Yes	Unsure	Yes	Yes
3 Adib-Hajbaghery & Etri, 2013	Unsure	Unsure	Yes	No	No	Yes	Yes	Yes	Yes	Unsure	NA	Yes
4 Atrian, et al. 2013	Yes	Yes	Unsure	Yes	Yes	Yes	No	Yes	Yes	Unsure	Unsure	Yes
5 Molassiotis, et al. 2013	Yes	Yes	Yes	Unsure	Unsure	Yes	No	Yes	Yes	Yes	Unsure	Yes
6 Noroozinia, et al. 2013	Unsure	Unsure	Yes	Yes	Yes	Yes	Yes	Yes	Yes	Unsure	NA	Yes
7 Sehhatie-Shafaie, et al. 2013	Yes	Yes	Yes	No	Yes	Yes	Yes	Yes	Yes	Unsure	NA	Yes
8 Chao, et al. 2013	Unsure	Unsure	Unsure	No	Yes	Yes	Unsure	Yes	Yes	Yes	Unsure	Yes
9 Soltanzadeh, et al. 2012	Yes	Unsure	Yes	Unsure	Unsure	Yes	Yes	Yes	Yes	Unsure	Unsure	Yes
10 Valiee, et al. 2012	Yes	No	Unsure	No	Yes	Yes	Yes	Yes	Yes	Unsure	NA	Yes
11 Suh, 2012	Yes	Yes	Unsure	Unsure	Unsure	Yes	No	Yes	Yes	Unsure	Unsure	Yes
12 McFadden, et al. 2012	Unsure	Unsure	Yes[1]	No	Yes[1]	Unsure	No	Yes	Yes	Yes	NA	Yes
13 Rad, et al. 2012	Unsure	Unsure	Yes	No	Yes	Yes	No	Yes	Yes	Yes	Unsure	Yes
14 White, et al. 2012	Yes	Unsure	Yes	Unsure	Yes	Yes	Yes	Yes	Yes	Yes	Yes	Yes
15 Hamidzadeh, et al. 2012	Unsure	Unsure	Unsure	No	Yes	Yes	Yes	Yes	Yes	Unsure	NA	Yes
16 Alessandrini, et al. 2012	Yes	Unsure	Yes	Yes	Yes	Yes	Yes	Yes	Unsure	Yes	NA	Yes
17 Soltani, et al. 2011	Yes	Unsure	Yes	Unsure	Yes	Yes	Yes	Yes	Yes	Yes	Unsure	Yes
18 Chang, et al. 2011	Yes	Unsure	Yes	No	No	Yes	Yes	Yes	Yes	Unsure	Unsure	Yes
19 McFadden, et al. 2011	Yes	Yes	Yes	No	Yes	Yes	No	Yes	Yes	Unsure	Unsure	Yes
20 Kashefi, et al. 2011	Yes	No	Yes	No	Yes	Yes	No	Yes	Yes	Unsure	Unsure	Yes
21 Bao, et al. 2011	Yes	No	Yes	No	Yes	Yes	No	Yes	Yes	Yes	NA	Yes
22 Majholm & Møller 2011	Yes	Yes	Yes	No	Yes	Yes	No	Yes	Yes	Yes	Unsure	Yes
23 Sinha, et al. 2011	Yes	Yes	Yes	Yes	Yes	Yes	Yes	Yes	Yes	Yes	Yes	Yes
24 Mirbagher-Ajorpaz, et al. 2011	Yes	Unsure	Yes	No	Yes	Yes	Yes	Yes	Yes	Yes	NA	Yes
25 Reza, et al. 2010	Unsure	Unsure	Yes	Yes	Yes	Yes	No	Yes	Yes	Yes	Unsure	Yes
26 Sun, et al. 2010	Yes	Yes	Unsure	No	Yes	Yes	Yes	Yes	Yes	Unsure	Unsure	Yes
27 Kashanian & Shahali 2010	Yes	Yes	Unsure	No	Unsure	Yes	Yes	Yes	Yes	Unsure	NA	Yes
28 Hjelmstedt, et al. 2010	Yes	Yes	Unsure	No	Yes[2]	Yes	No	Yes	Yes	Unsure	NA	Yes
29 McFadden & Hernández 2010	Yes	Yes	Yes	No	Yes	Yes	No	Yes	Unsure	Unsure	Unsure	Yes
30 Nordio & Romanelli 2008	Yes	Yes	Yes	Yes	Unsure	Yes	No	Yes	Unsure	Unsure	Unsure	Yes
31 Wang, et al. 2008	Yes	Unsure	Yes	No	Yes	Yes	Yes	Yes	Yes	Yes	NA	Yes
32 Maa, et al. 2007	Yes	Unsure	Unsure	No	Unsure	Yes	No	Yes	Yes	Yes	Unsure	Yes
33 Turgut, et al. 2007	Yes	Yes	Unsure	Yes	Yes	Yes	No	Yes	Yes	Yes	Unsure	Yes
34 Shin, et al. 2007	Yes	Unsure	Yes	No	Unsure	Unsure	Unsure	Yes	Yes	Yes	Unsure	Yes
35 Lang, et al. 2007	Yes	Yes	Yes	No	Yes	Yes	Yes	Yes	Yes	Unsure	NA	Yes
36 Wu, et al. 2007	Unsure	Unsure	Yes	No	Yes	Yes	No	Yes	Yes	Yes	Unsure	Yes
37 Heazell, et al. 2006	Yes	Yes	Yes	Unsure	Unsure	Yes	No	Yes	Yes	Yes	Yes	Unsure
38 Hsu, et al. 2006	Yes	Unsure	Unsure	No	Unsure	Yes	Yes	Yes	Yes	Unsure	Unsure	Yes
39 Ho, et al. 2006	Yes	Yes	Unsure	Unsure	Yes	Yes	Yes	Yes	Yes	Yes	Unsure	Yes
40 Wang, et al. 2005	Yes	Unsure	Unsure	No	Yes	Unsure	Unsure	Yes	Yes	Yes	NA	Yes
41 Alkaissi, et al. 2005	Yes	Yes	Yes[1]	Unsure	Yes[1]	Yes	Yes	Yes	Yes	Yes	NA	No
42 Klein, et al. 2004	Yes	Unsure	Yes	Yes	Yes	Yes	Yes	Yes	Yes	Yes	Unsure	Yes
43 Wu, et al. 2004	Unsure	Unsure	Yes	No	Yes	Yes	No	Yes	Yes	Yes	Unsure	Yes
44 Lee, et al. 2004	Unsure	Unsure	Yes	No	Yes	Yes	No	Yes	Yes	Yes	NA	Yes
45 Bertalanffy, et al. 2004	Yes	Yes	Unsure	No	Yes	Yes	Yes	Yes	Yes	Unsure	NA	Yes
46 Chen, et al. 2003	Yes	Unsure	Yes	No	Unsure	Yes	Yes	Yes	Yes	Yes	Unsure	Yes
47 Tsay & Chen, 2003	Unsure	Unsure	Yes	No	Yes	Yes	Unsure	Yes	Yes	Yes	Unsure	Yes
48 Dent, et al. 2003	Yes[3]	Yes[3]	Unsure	Unsure	Unsure	Yes	Yes	Yes	Unsure	Yes	Unsure	Yes
49 Schultz, et al. 2003	Yes	Unsure	Unsure	No	Unsure	No	No	Yes	Yes	Yes	Yes	Yes
50 Samad, et al. 2003	Yes	Unsure	Unsure	Unsure	Yes	Yes	Yes	Yes	Yes	Yes	Unsure	Yes
51 Kober, et al. 2002	Yes	Unsure	Yes	No	Yes	Yes	Yes	Yes	Yes	Yes	NA	Yes
52 Alkaissi, et al. 2002	Yes	Yes	Yes[4]	Yes[4]	Yes[4]	Yes	No	Yes	Unsure	Yes	Unsure	Yes
53 Norheim, et al. 2001	Unsure	Unsure	Unsure	Yes	Unsure	Yes	Yes	Yes	Yes	Unsure	Unsure	Yes
54 Steele, et al. 2000	Yes	Yes	Yes	No	Yes	Yes	No	Yes	Yes	No	Unsure	Yes
55 Agarwal, et al. 2000	Yes	Unsure	Yes	Unsure	Yes	Yes	Yes	Yes	Yes	Yes	Unsure	Yes
56 Harmon, et al. 2000	Unsure	Unsure	Yes	Unsure	Yes	Unsure	Yes	Yes	Yes	Yes	Unsure	Yes
57 Harmon, et al. 1999	Yes	Yes	Yes	Yes	Yes	Yes	Yes	Yes	Yes	Yes	NA	Yes
58 Alkaissi, et al. 1999	Unsure	Unsure	Unsure	Yes[5]	Yes	Yes	No	Yes	Yes	Yes	Unsure	Yes
59 Woods, 1999	Yes	Unsure	Yes	Yes	Yes	Yes	Yes	No	Unsure	Yes	Unsure	Yes
60 Duggal, et al. 1998	Yes	Unsure	Yes	Yes	Yes	Yes	No	Yes	Yes	Yes	Yes	Yes
61 Fan, et al. 1997	Unsure	Unsure	Unsure	Yes	Yes	Yes	Yes	Yes	Yes	Yes	Unsure	Yes
62 Felhendler & Lisander 1996	Yes	Yes	Unsure	Unsure	Yes	Unsure	Unsure	Yes	Yes	Yes	NA	Yes
63 O'Brien, et al. 1996	Yes	Yes	Yes[6]	Unsure	Yes	Yes	No	Yes	Unsure	No	Unsure	Yes
64 Belluomini, et al. 1994	Unsure	Unsure	Yes	Yes	Yes	Yes	No	Yes	Yes	Yes	Unsure	Yes
65 Bayreuther, et al. 1994	Yes	Yes	Yes	Yes	Yes	No	No	Yes	No	Unsure	Unsure	Yes
66 Lewis, et al. 1991	Unsure	Unsure	Yes	No	Yes	Yes	No	Yes	Yes	Yes	Yes	Yes

Item 1: "Was the method of randomization adequate?", **Item 2**: "Was the treatment allocation concealed?", **Item 3**: "Was the patient blinded to the intervention?", **Item 4**: "Was the care provider blinded to the intervention?", **Item 5**: "Was the outcome assessor blinded to the intervention?", **Item 6**: "Was the drop-out rate described and acceptable?", **Item 7**: "Were all randomized participants analyzed in the group to which they were allocated?", **Item 8**: "Are reports of the study free of suggestion of selective outcome reporting?", **Item 9**: "Were the groups similar at baseline regarding the most important prognostic indicators?", **Item10**: "Were co-interventions avoided or similar?", **Item 11**: "Was the compliance acceptable in all groups?", **Item 12**: "Was the timing of the outcome assessment similar in all groups?"

1 Three groups were included in this study, but blinding of patients and outcome assessors was only applicable for true acupressure and sham/placebo acupressure groups.

2 Three groups were included in this study, but blinding of outcome assessors was only applicable for true acupressure and sham/placebo acupressure groups.

3 Randomization was only applicable for true acupressure and sham/placebo acupressure groups.

4 Three groups were included in this study, but blinding of patients, care providers and outcome assessors was only applicable for true acupressure and sham/placebo acupressure groups.

5 Three groups were included in this study, but blinding of care providers was only applicable for true acupressure and sham/placebo acupressure groups.

6 Three groups were included in this study, but blinding of patients was only applicable for true acupressure and sham/placebo acupressure groups.
